# One-pot synthesis of 2-substituted 4*H*-3,1-benzoxazin-4-one derivatives under mild conditions using iminium cation from cyanuric chloride/dimethylformamide as a cyclizing agent

**DOI:** 10.1186/1752-153X-7-58

**Published:** 2013-03-27

**Authors:** Mehdi Shariat, Mohd Wahid Samsudin, Zuriati Zakaria

**Affiliations:** 1 School of Chemical Science and Food Technology, Faculty of Science and Technology, University Kebangsaan Malaysia (UKM), 43600, Selangor, Malaysia; 2 Department of Chemistry, Faculty of Science, Payame Noor University of Golpayegan (PNU), 87117-43153, Isfahan, Iran; 3 Malaysia Japan International Institute of Technology, University Technology Malaysia (UTM), 54100, Kuala Lumpur, Malaysia

**Keywords:** Iminium cation, Benzoxazinone, Cyclizing agent, Cyclodehydration

## Abstract

**Background:**

The derivatives of 2-substituted 4*H*-3,1-benzoxazin-4-one belong to a significant category of heterocyclic compounds, which have shown a wide spectrum of medical and industrial applications.

**Results:**

A new and effective one-pot method for the synthesis of 2-substituted 4*H*-3,1-benzoxazin-4-one derivatives is described in this paper. By using the iminium cation from a mixture of cyanuric chloride and dimethylformamide as a cyclizing agent, a series of 2-substituted 4*H*-3,1-benzoxazin-4-one derivatives was synthesized in high yield under mild conditions and simple workup.

**Conclusions:**

The iminium cation from a mixture of cyanuric chloride and *N,N*-dimethylformamide is an effective cyclizing agent for the room temperature one-pot synthesis of 2-substituted 4*H*-3,1-benzoxazin-4-one derivatives in high yields through a cyclodehydration reaction. Furthermore, the method was performed under mild conditions characterized by simplified pathways and workup, minimized energy, and fewer reaction steps, compared with the previous methods. The proposed method, which is a simpler alternative than the published methods, is applicable for the synthesis of other 2-substituted 4*H*-3,1-benzoxazin-4-one derivatives.

## Background

The derivatives of 2-substituted 4*H*-3,1-benzoxazin-4-one belong to a significant category of heterocyclic compounds that have shown a wide spectrum of medical and industrial applications. Some of them are used as an elastase inhibitor [1-3], anti-neoplastic agent, enzyme inhibitor [4], protease inhibitor, and fungicidal [5]. In addition, they are used as a starting material for the preparation of 2,3-disubstituted 4(3*H*)-quinazolinone derivatives, which are known to have medicinal properties.

**Scheme 1:**
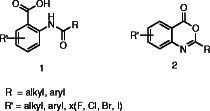
***N*-acylated anthranilic acid and 2-substituted-4*H*-3,1-benzoxazin-4-one.**

The majority of the reported methods for producing 2-substituted benzoxazin-4-one formula **2** are based on the preparation of *N*-acylated anthranilic acid derivative formula **1** as an intermediate from anthranilic acid derivatives and a chloride of a carboxylic acid (Scheme 1).

**Scheme 2:**
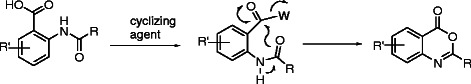
Formation of the benzoxazin-4-one rings.

The benzoxazin-4-one ring is formed from intermediate formula **1** and cyclizing agents, such as acetic anhydride [6-9], polyphosphoric acid [10], sulfuric acid [11], and pyridine [12], which can convert the OH of the carboxylic acid group into a good living group **W** to form the benzoxazin-4-one ring (Scheme 2).

Optimizing the previous methods or designing new routes according to the principles of green chemistry for the synthesis and workup of 4*H*-3,1-benzoxazin-4-one derivatives are essential given the increasing application of this group of fused heterocyclic compounds.

Green chemistry provides an interesting approach for the preparation and application of chemical compounds. This approach is presented as a set of twelve principles [13,14]. From the standpoint of green chemistry, chemists should establish a group of parameters for the ideal chemical synthesis design [14]. A simple pathway and workup, minimized energy, higher yield, lower reagent loss, and fewer reaction steps are among the important parameters in a green chemical pathway. Following this approach, the proposed method for the synthesis of 2-substituted-4*H*-3,1-benzoxazin-4-one derivatives under mild conditions is an attempt to design a greener reaction.

In this research, a series of 2-substituted-4*H*-3,1-benzoxazin-4-one derivatives was synthesized using the iminium cation from a mixture of cyanuric chloride and dimethylformamide as an effective cyclizing agent as well as the solvent as a catalyst, without the traditional heating or microwave irradiation.

Cyanuric chloride is a commercially available reagent that is commonly used by organic chemists. Numerous articles and reviews have reported various applications of cyanuric chloride in ordinary organic reactions. One of the common applications of cyanuric chloride is the conversion of carboxyl groups into active esters for the preparation of nitrile, acyl azide, ester, amide, and acyl chloride from carboxylic acid derivatives [15,16]. Furthermore, cyanuric chloride is used to prepare alkyl chlorides from alcohols [17], amides from ketoximes [18], nitrile from aldoxime [19], isonitrile from formamide [20], disulfide from dimethylsulfoxide [21], and cyclic lactones [22]. In addition, the mixture of cyanuric chloride and dimethylformamide is used as a new organic reagent for the conversion of a broad sequence of secondary and primary alcohols to the corresponding alkyl chlorides and iodides [23,24]. Various ketoximes prepared from the related ketones undergo the Beckmann rearrangement upon reaction with a mixture of cyanuric chloride/dimethylformamide [24]. Moreover, the mixture of cyanuric chloride/dimethylformamide is used for the conversion of *β*-amino alcohols to the corresponding chlorides [25].

## Results and discussions

A series of 2-substituted 4*H*-3,1-benzoxazine-4-one formulae **2a-i** was prepared in good yield at room temperature using anthranilic acid and nine different derivatives of acyl chloride and benzoyl chloride as starting materials as well as iminium cation as cyclizing agent in dimethylformamide in the presence of triethylamine (Table 1).

**Table 1:** **Products, yields, and melting points related to Scheme **3

**No.**	**Product**	**Yield (%)**	**mp (T/ **^**o**^**C)**	**Lit. mp (T/**^**o**^**C)**
**2a**		86	123-124	123-125 [26]
**2b**		89	236-238	
**2c**		78	106-108	
**2d**		79	89-91	
**2e**		82	261-263	
**2f**		89	184-185	184-185 [27,28]
**2g**		80	148-149	147-148 [29]
**2h**		83	167-168	
**2i**		86	202-204	203 [27]

**Scheme 3:**
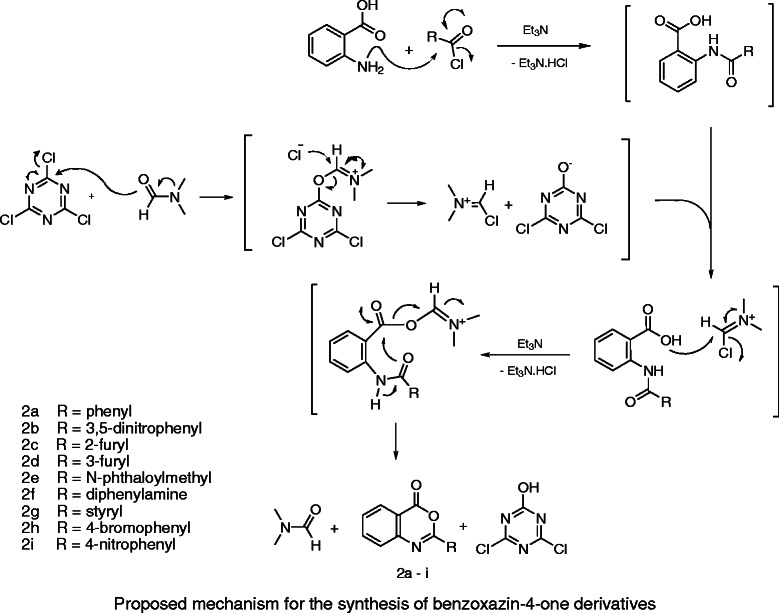
Proposed mechanism for the synthesis of benzoxazin-4-one derivatives.

During the reaction, *N*-acylated anthranilic acid is produced as an intermediate **1** from anthranilic acid and a chloride of alkyl or aryl carboxylic acid in the presence of triethylamine as the HCl scavenger via *N*-acylation reaction. Given the special structure of *N*-acylated anthranilic acid, benzoxazin-4-one ring can be formed by the intramolecular nucleophilic attack in intermediate **1**. However, this process is not generally thought of as viable because of the low electrophilicity of the amide group. Instead, the cyclization reaction can be performed to induce the conversion of carboxylic acid group in *N*-acylated anthranilic acid into an active ester using a cyclizing agent and traditional heating or microwave irradiation. In this research, the iminium cation from a mixture of cyanuric chloride and dimethylformamide acted as a cyclizing agent at room temperature. The 2-substituted 4*H*-3,1-benzoxazin-4-one derivatives were prepared at room temperature and ambient pressure under mild conditions (Scheme 3).

The positive charge, size, and planar skeleton of the iminium cation make it an easy target for the OH group, which could result in the production of activated ester in a short time with good yield. However, the stability of benzoxazin-4-one ring provides an appropriate condition for a mild cyclodehydration reaction as the result of the cyclization reaction and the role of dimethylformamide as a good living group in the cyclizing step.

In published papers and reviews, acetic anhydride is commonly used as a cyclizing agent for the synthesis of benzoxazin-4-one derivatives because it can be used in simpler conditions compared with the other known cyclizing agents. However, the use of this compound as a cyclizing agent has several disadvantages. For example, acetic anhydride normally works as a cyclizing agent through traditional heating or microwave irradiation. In addition, this compound is listed as a United State Drug Enforcement Administration (U. S. DEA List II precursor), and restricted in numerous countries. On the contrary, the iminium cation can be a good cyclizing agent in cyclodehydration reactions because it can be used at room temperature with commercially available cyanuric chloride and dimethylformamide.

## Conclusions

The iminium cation obtained from a mixture of cyanuric chloride and *N,N*-dimethylformamide is an effective reagent for the room temperature one-pot synthesis of 2-substituted 4*H*-3,1-benzoxazin-4-one derivatives in high yields through cyclodehydration. The proposed method can be performed under mild conditions, with simplified pathways and workup, minimized energy, and fewer reaction steps, compared with the reported methods. Moreover, this method is applicable for the synthesis of other 2-substituted 4*H*-3,1-benzoxazin-4-one derivatives.

## Methods

### General

The structures of products **2a-i** were confirmed by analysis using spectral data (^1^H-NMR, ^13^C-NMR, FT-IR and HRMS). All spectral data and spectrum are represented in Additional file 1. The FT-IR spectra were measured by using KBr pellets. The NMR spectra were recorded on 600 MHz spectrometer and chemical shifts are reported relative to TMS. The mass spectra were recorded using a TOF-Q instrument was operated in positive ion mode. The melting points were measured in open capillary tubes without correction. All commercial reagents were synthesis grade and were used as received without additional purification. The procedures for preparation and purification of reported products are the same.

### Preparation of 2-phenyl-4*H*-3,1-benzoxazin-4-one (2a)

Benzoyl chloride (0.349 ml, 3 mmol) was added to a stirred solution of anthranilic acid (0.411 g, 3 mmol) and triethylamine (0.460 mL, 3.3 mmol) in chloroform (10 mL). The mixture was stirred at room temperature for 2 hours, then, a yellow light colour solution of Cyanuric chloride (0.553 g, 3 mmol) in DMF (5 mL) was added to the stirred mixture. After 4 hours, the solvent was evaporated in vacuum and the residual was poured into distilled water (20 mL) and ice. Then, the filtrated solids were washed with a saturated solution of NaHCO_3_ (10 mL, two times) and distilled water (two times, 25 mL each). The white precipitate was recrystallized from a 1:1 diethyl ether/ethanol mixture to give the fine needle crystal of **2a** (0.578 gr, yield: 86%, mp: 123–124°C). IR (KBr): *v*_max_/cm^−1^ 1764, 1622, 1603, 1541, 1266, 1077. ^1^H-NMR (DMSO): δ ppm 8.20 (ddd, *J* = 7.2, 1.2, 0.6 Hz, 2H), 8.15 (ddd, *J* = 7.2, 1.2, 0.6 Hz 1H), 7.95 (tdd, *J* = 8.4, 7.2, 1.2 Hz, 1H), 7.59-7.73 (m, 5H). ^13^C-NMR (DMSO): 159.4, 156.8, 146.7, 137.3, 133.2, 130.5, 129.5, 129.1, 128.5, 128.3, 127.4, 117.4. HRMS (ESI-TOF) *m/z*: [M+H]^+^ Calcd for C_14_H_10_NO_2_ (224.0707); Found 224.0706.

### Preparation of 2-(3,5-dinitrophenyl)-4*H*-3,1-benzoxazin-4-one (2b)

3,5-dinitrobenzoyl chloride (0.692 gr, 3 mmol) is used for preparation of **2b** using the same procedure as **2a**. The sea-urchin shaped crystal was prepared (0.839 gr, yield: 89%, mp: 236–238°C) from crystallization of the gray powder of **2b** in 1:1 ether/ethanol. IR (KBr): *v*_max_/cm^−1^ 1776, 1622, 1603, 1541, 1473, 1266, 1077. ^1^H-NMR (DMSO): δ ppm 9.13 (dd, *J* = 1.8, 1.2 Hz, 2H), 9.04 (dd, *J* = 1.4, 1.2 Hz, 1H), 8.23 (ddd, *J* = 7.8, 1.2, 0.6 Hz, 1H), 8.02 (ddd, *J* =8.4, 7.8,1.2 Hz, 1H), 7.90 (ddd, *J* =7.8, 1.2, 0.6 Hz, 1H) and 7.73 (ddd, *J* = 8.4, 7.8,1.2 Hz, 1H). ^13^C-NMR (DMSO): 158.6, 153.6, 149.1, 145.9, 137.7, 133.7, 130.2, 128.8, 127.9, 127.6, 122.1, 117.8. HRMS (ESI-TOF) *m/z*: [M+H]^+^ Calcd for C_14_H_8_N_3_O_6_ (314.0408); Found 314.0390.

### Preparation of 2-(furan-2-yl)-4*H*-3,1-benzoxazin-4-one (2c)

For preparation of **2c,** 2-Furoyl chloride was used by using the same procedure, as **2a**. The product was isolated as a white crystalline solid (0.500 gr, yield: 78%, mp 106–108°C)_**.**_ IR (KBr): *v*_max_/cm^−1^ 1753, 1683, 1604, 1557, 1256, 1011. ^1^H-NMR (DMSO, δ ppm): 8.12 (ddd, *J* = 7.8, 1.2, 0.6 Hz, 1H), 8.09 (dd, *J* = 0.6, 1.8 Hz, 1H), 7.93 (ddd, *J* = 0.6, 1.2, 7.8 Hz, 1H), 7.66 (ddd, *J* = 7.8, 1.2, 7.8 Hz, 1 H), 7.59 (ddd, *J* = 7.8, 1.2, 7.8 Hz, 1H), 7.45 (dd, *J* = 3.6, 0.6 Hz, 1H), 6.80 (dd, *J* = 1.8, 3.6 Hz, 1H). ^13^C-NMR (DMSO): 158.7, 149.6, 148.4, 146.7, 144.6, 137.4, 128.8, 128.6, 127.1, 117.5, 113.4. HRMS (ESI-TOF) *m/z*: [M+H]^+^ Calcd for C_12_H_8_NO_3_ (214.0499); Found 214.0497.

### Preparation of 2-(furan-3-yl)-4*H*-3,1-benzoxazin-4-one (2d)

3-Furoyl chloride was used for preparation **2d** and the product was isolated as a white crystalline solid (0.506 gr, yield: 79%, mp 89–91°C). IR (KBr): *v*_max_/cm^−1^ 1764, 1644, 1632, 1603, 1258, 1069. ^1^H-NMR (DMSO, δ ppm): 8.55 (dd, *J* = 1.8, 0.6 Hz, 1H), 8.16 (ddd, *J* = 7.8, 1.2, 0.6 Hz, 1H), 7.92 (ddd, *J* = 7.8, 1.2, 0.6 Hz, 1H), 7.89 (dd, *J* = 1.8, 1.2 Hz, 1H), 7.64 (ddd, *J* = 7.8, 1.2, 0.6 Hz, 1H), 7.59 (ddd, *J* = 7.4, 7.2, 1.2 Hz, 1 H), 7.00 (dd, *J* = 1.8, 0.6 Hz, 1 H). ^13^C-NMR (DMSO): 159.2, 153.5, 147.4, 146.7, 145.9, 137.4, 128.8, 128.6, 126.93, 119.8, 117.3, 109.2. HRMS (ESI-TOF) *m/z*: [M+H]^+^ Calcd for C_12_H_8_NO_3_ (214.0499); Found 214.0478.

### Preparation of 2-(*N*-phthaloylmethyl)-4*H*-3,1-benzoxazin-4-one (2e)

Benzoxazinone **2e** was prepared from phthalimidoacetyl chloride (0.671 gr, 3 mmol) the same procedure as above and product was collected as a light yellow crystalline solid (0.752 gr, yield: 82%, mp 261–263°C). IR (KBr): *v*_max_/cm^−1^ 1776, 1688, 1591, 1529, 1258, 1088. ^1^H-NMR (DMSO): δ ppm 7.89-8.08 (m, 5 H, Ar-H), 7.87 (ddd, *J* = 7.8, 1.2, 0.6 Hz 1H, Ar-H), 7.60 (ddd, *J* = 7.8, 1.2, 6.6 Hz, 1 H, Ar-H), 7.23 (ddd*, J* = 6.6, 7.2, 1.2 Hz, 1 H, Ar-H), 3.77 (s, 2 H, CH_2_). ^13^C-NMR (DMSO): 167.9, 167.8, 165.7, 138.6, 135.2, 134.2, 132.0, 131.0, 124.5, 124.4, 122.3, 119.8, 52.7. HRMS (ESI-TOF) *m/z*: [M+H]^+^ Calcd for C_17_H_11_N_2_O_4_ (307.0713); Found 307.0720.

### Preparation of 2-(4-bromophenyl)-4*H*-3,1-benzoxazin-4-one (2f)

Final product was isolated as a crystalline solid (0.802 gr, yield: 89%, mp: 184–185°C). IR (KBr): *v*_max_/cm^−1^ 1762, 1619, 1602, 1586, 1256, 1067. ^1^H-NMR (DMSO): δ ppm 8.16 (ddd, *J* = 7.2, 1.2, 0.6 Hz, 1H), 8.11 (ddd, *J* = 6.6, 1.8, 0.6 Hz, 2H), 7.96 (ddd, *J =* 8.4, 7.2, 1.2 Hz, 1H), 7.81 (ddd, *J* = 6.0, 1.2, 0.6 Hz, 2H), 7.73 (ddd, *J =* 8.4, 1.2, 0.6 Hz, 1H) and 7.64 (ddd, *J* = 7.8, 7.2, 0.6 Hz, 1H). ^13^C-NMR (DMSO): 159.2, 156.2, 154.1, 137.4, 132.6, 130.2, 129.8, 129.2, 128.6, 127.4, 127.1, 117.5. HRMS (ESI-TOF) *m/z*: [M+H]^+^ Calcd for C_14_H_9_BrNO_2_ (301.9811); Found 301.9807.

### Preparation of 2-(styryl)-4*H*-3,1-benzoxazin-4-one (2g)

The product was collected as a yellow crystalline solid (0.600 gr, yield: 80%, mp: 148–149°C). IR (KBr): *v*_max_/cm^−1^ 1761, 1635, 1592, 1566, 1251, 1040. ^1^H-NMR (DMSO): δ ppm 7.78 (d, *J* = 16.2 Hz, 1H), 7.59 - 8.16 (m, 9H, Ar-H), 7.01 (d, *J* = 16.2 Hz, 1H). ^13^C-NMR (DMSO): 159.2, 157.3, 147.1, 141.6, 137.3, 134.9, 130.8, 129.4, 128.9, 128.7, 128.5, 127.1, 119.7, 117.3. HRMS (ESI-TOF) *m/z*: [M+H]^+^ Calcd for C_16_H_12_NO_2_ (250.0863); Found 250.0851.

### Preparation of 2-(diphenylamino)-4*H*-3,1-benzoxazin-4-one (2h)

The result was isolated as a crystalline solid (83% yield, 0.780 gr, mp: 167–168°C). IR (KBr): *v*_max_/cm^−1^ 1745, 1619, 1582, 1489, 1267, 1072. ^1^H-NMR (DMSO): δ ppm 7.93 (ddd, *J =* 8.4, 1.8, 0.6 Hz, 1H), 7.69 (ddd, *J =* 8.4, 7.2, 1.8 Hz, 1H), 7.42-7.45 (m, 8 H, Ar-H), 7.29 - 7.32 (m, 2H, Ar-H), 7.26 (ddd, *J =* 7.8, 7.2, 0.6 Hz, 1H), 7.16 (ddd, *J =* 7.8, 1.2, 0.6 Hz, 1H). ^13^C-NMR (DMSO): 159.4, 153.1, 150.1, 142.7, 137.2, 128.5, 129.7, 128.0, 127.2, 125.1, 124.9, 114.1. HRMS (ESI-TOF) *m/z*: [M+H]^+^ Calcd for C_20_H_15_N_2_O_2_ (315.1128); Found 315.1131.

### Preparation of 2-(4-nitrophenyl)-4*H*-3,1-benzoxazin-4-one (2i)

The procedure **2a** was used for synthesis and workup of 2-(4-nitrophenyl)-4*H*-3,1-benzoxazin-4-one and the final product was gathered as a yellow wish crystalline solid (0.779 gr, yield: 86%, mp: 202–204). IR (KBr): *v*_max_/cm^−1^ 1766, 1607, 1589, 1522, 1493, 1251, 1082. ^1^H-NMR (DMSO): δ ppm 8.43 (ddd, *J* = 6.6, 1.8, 0.6 Hz, 2 H), 8.39 (ddd, *J* = 0.6, 7.8, 1.1 Hz, 1H), 8.18 (ddd, *J* = 0.6, 7.2, 1.2 Hz, 2H), 8.00 (ddd, *J* = 7.8, 1.2, 0.6 Hz, 1H), 7.71 (ddd, *J* = 8.4, 1.2, 7.2 Hz, 1H), 7.30 (ddd, *J* = 7.2, 7.8, 0.6 Hz, 1H). ^13^C-NMR (DMSO): 159.0, 156.2, 150.3, 146.7, 137.4, 136.4, 130.1, 129.8, 128.6, 127.2, 125.4, 119.1. HRMS (ESI-TOF) *m/z*: [M+H]^+^ Calcd for C_14_H_9_N_2_O_4_ (269.0557); Found 269.0554.

## Competing interests

This research focused on an application of iminium cation, namely, as a cyclizing agent in the cyclodehydration reaction under mild conditions. Iminium cation may be usable in other reactions in this category. In addition, the role of dimethylformamide was interesting because it acted as a catalyst.

## Authors’ contributions

MS proposed the subject, designed the study, helped in the results and discussion, and carried out the synthesis of all the products. MWS and ZZ conceived the study and participated in its design, results and discussion, and coordination, as well as helped draft the manuscript. All the authors read and approved the final manuscript.

## Supplementary Material

Additional file 1 **The spectral data (**^**1**^**H-NMR, **^**13**^**C-NMR, FT-IR and HRMS) for products 2a-i are represented in Additional file 1.** Click here for file
