# Supplementary material for: One-pot synthesis of 2-substituted 4H-3,1-benzoxazin-4-one derivatives under mild conditions using iminium cation from cyanuric chloride/dimethylformamide as a cyclizing agent
Source: Chem Cent J. 2013 Mar 27;7:58. doi: 10.1186/1752-153X-7-58 (PMC3662593; doi:10.1186/1752-153X-7-58)
Supplement: Additional file 1 — The spectral data (1H-NMR, 13C-NMR, FT-IR and HRMS) for products 2a-i are represented in Additional file 1. [file 1752-153X-7-58-S1.docx]

**One-Pot Synthesis of 2-Substituted 4*H*-3,1-Benzoxazin-4-One Derivatives Under Mild Condition, Using Iminium Cation From Cyanuric Chloride/Dimethylformamide as a Cyclizing Agent**

Mehdi Shariat^1,2^*, Mohd Wahid Samsudin^1^, Zuriati Zakaria^3^

^1^ School of Chemical Science and Food Technology, Faculty of Science and Technology, University Kebangsaan Malaysia (UKM), 43600 Selangor, Malaysia.

^2^ Department of Chemistry, Faculty of Science, Payame Noor University of Golpayegan (PNU), 87117-43153 Isfahan, Iran.

^3^ Malaysia Japan International Institute of Technology, University Technology Malaysia (UTM), 54100 Kuala Lumpur, Malaysia.

* Corresponding author

Email addresses:

Mehdi Shariat: [sm.shariat@yahoo.com](mailto:sm.shariat@yahoo.com)

Mohd Wahid Samsudin: [wahid@ukm.my](mailto:wahid@ukm.my)

Zuriati Zakaria: [zuriz@ic.utm.my](mailto:zuriz@ic.utm.my)

**Table of Contents**

[1. The IR, 1H-NMR, 13C-NMR and HRMS spectra of product No. 2a 4](#_Toc345233686)

[‎2.1 IR spectrum (KBr) of **2a** 4](#_Toc345233687)

[‎2.2 ^1^H-NMR spectrum of **2a** 5](#_Toc345233688)

[‎2.3 ^13^C-NMR spectrum of **2a** 6](#_Toc345233689)

[‎2.4 HRMS spectrum of **2a** 7](#_Toc345233690)

[2. The IR, 1H-NMR, 13C-NMR and HRMS spectra of product No. 2b 8](#_Toc345233691)

[‎3.1 IR spectrum (KBr) of **2b** 8](#_Toc345233692)

[‎3.2 HRMS spectrum of **2b** 9](#_Toc345233693)

[‎3.3 ^1^H-NMR spectrum of **2b** 10](#_Toc345233694)

[‎3.4 Enlarged ^1^H-NMR spectrum of **2b** 11](#_Toc345233695)

[‎3.5 ^13^C-NMR spectrum of **2b** 12](#_Toc345233696)

[3. The IR, 1H-NMR, 13C-NMR and HRMS spectra of product No. 2c 13](#_Toc345233697)

[‎4.1 IR spectrum of **2c** 13](#_Toc345233698)

[‎4.2 HRMS spectrum of **2c** 14](#_Toc345233699)

[‎4.3 ^1^H-NMR spectrum of **2c** 15](#_Toc345233700)

[‎4.4 Enlarged ^1^H-NMR spectrum of **2c** 16](#_Toc345233701)

[‎4.5 ^13^C-NMR spectrum of **2c** 17](#_Toc345233702)

[4. The IR, 1H-NMR, 13C-NMR and HRMS spectra of product No. 2d 18](#_Toc345233703)

[‎5.1 IR spectrum of **2d** 18](#_Toc345233704)

[‎5.2 HRMS spectrum of **2d** 19](#_Toc345233705)

[‎5.3 ^1^H-NMR spectrum of **2d** 20](#_Toc345233706)

[‎5.4 Enlarged ^1^H-NMR spectrum of **2d** 21](#_Toc345233707)

[‎5.5 ^13^C-NMR spectrum of **2d** 22](#_Toc345233708)

[5. The IR, 1H-NMR, 13C-NMR and HRMS spectra of product No. 2e 23](#_Toc345233709)

[‎6.1 IR spectrum of **2e** 23](#_Toc345233710)

[‎6.2 HRMS spectrum of **2e** 24](#_Toc345233711)

[‎6.3 ^1^H-NMR specrum of **2e** 25](#_Toc345233712)

[‎6.4 Enlarged H-NMR spectrum of **2e** 26](#_Toc345233713)

[‎6.5 Enlarged ^13^C-NMR spectrum of **2e** 27](#_Toc345233714)

[6. The IR, 1H-NMR, 13C-NMR and HRMS spectra of product No. 2f 28](#_Toc345233715)

[‎7.1 FT-IR spectrum of **2f** 28](#_Toc345233716)

[‎7.2 HRMS spectrum of **2f** 29](#_Toc345233717)

[‎7.3  ^1^H-NMR of **2f** 30](#_Toc345233718)

[‎7.4 Enlarged ^1^H-NMR of **2f** 31](#_Toc345233719)

[‎7.5 ^13^C-NMR of **2f** 32](#_Toc345233720)

[‎7.6 Enlarged ^13^C-NMR of **2f** 33](#_Toc345233721)

[7. The IR, 1H-NMR, 13C-NMR and HRMS spectra of product No. 2g 34](#_Toc345233722)

[‎8.1 IR spectrum of **2g** 34](#_Toc345233723)

[‎8.2 HRMS (MS-MS) of **2g** 35](#_Toc345233724)

[‎8.3 ^1^H-NMR spectrum of **2g** 36](#_Toc345233725)

[‎8.4 Enlarged ^1^H-NMR spectrum of **2g** 37](#_Toc345233726)

[‎8.5 ^13^C-NMR spectrum of **2g** 38](#_Toc345233727)

[‎8.6 Enlarged ^13^C-NMR spectrum of **2g** 39](#_Toc345233728)

[8. The IR, 1H-NMR, 13C-NMR and HRMS spectra of product No. 2h 40](#_Toc345233729)

[‎9.1 FT-IR spectrum of **2h** 40](#_Toc345233730)

[‎9.2 HRMS spectrum of **2h** 41](#_Toc345233731)

[‎9.3 ^1^H-NMR spectrum of **2h** 42](#_Toc345233732)

[‎9.4 Enlarged ^1^H-NMR spectrum of **2h** 43](#_Toc345233733)

[‎9.5 ^13^C-NMR spectrum of **2h** 44](#_Toc345233734)

[9. The IR, 1H-NMR, 13C-NMR and HRMS spectra of product No. 2i 45](#_Toc345233735)

[‎10.1 FT-IR spectrum of **2i** 45](#_Toc345233736)

[‎10.2 HRMS spectrum of **2i** 46](#_Toc345233737)

[‎10.3 ^1^H-NMR spectrum of **2i** 47](#_Toc345233738)

[‎10.4 Enlarged ^1^H-NMR spectrum of **2i** 48](#_Toc345233739)

[‎10.5 ^13^C-NMR spectrum of **2i** 49](#_Toc345233740)

# The IR, 1H-NMR, 13C-NMR and HRMS spectra of product No. 2a


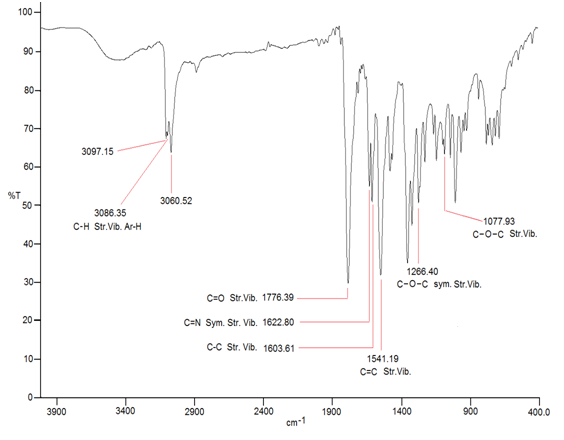


## ‎1.1 IR spectrum (KBr) of **2a**


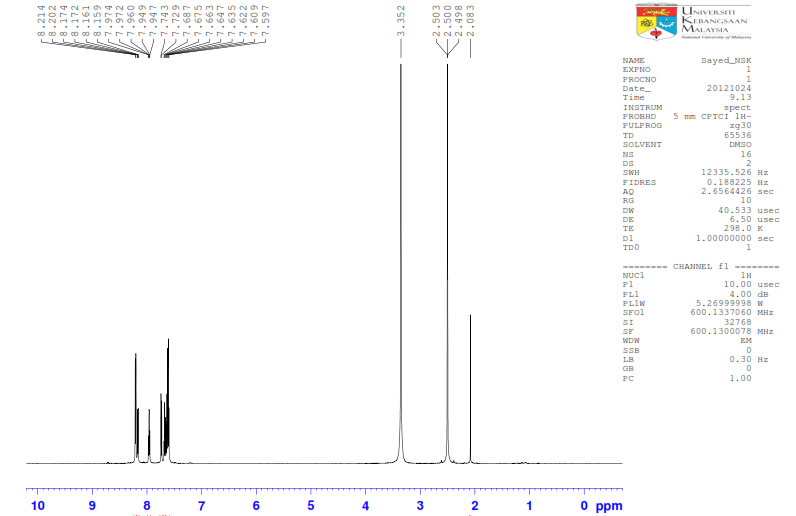


## ‎1.2 ^1^H-NMR spectrum of **2a**


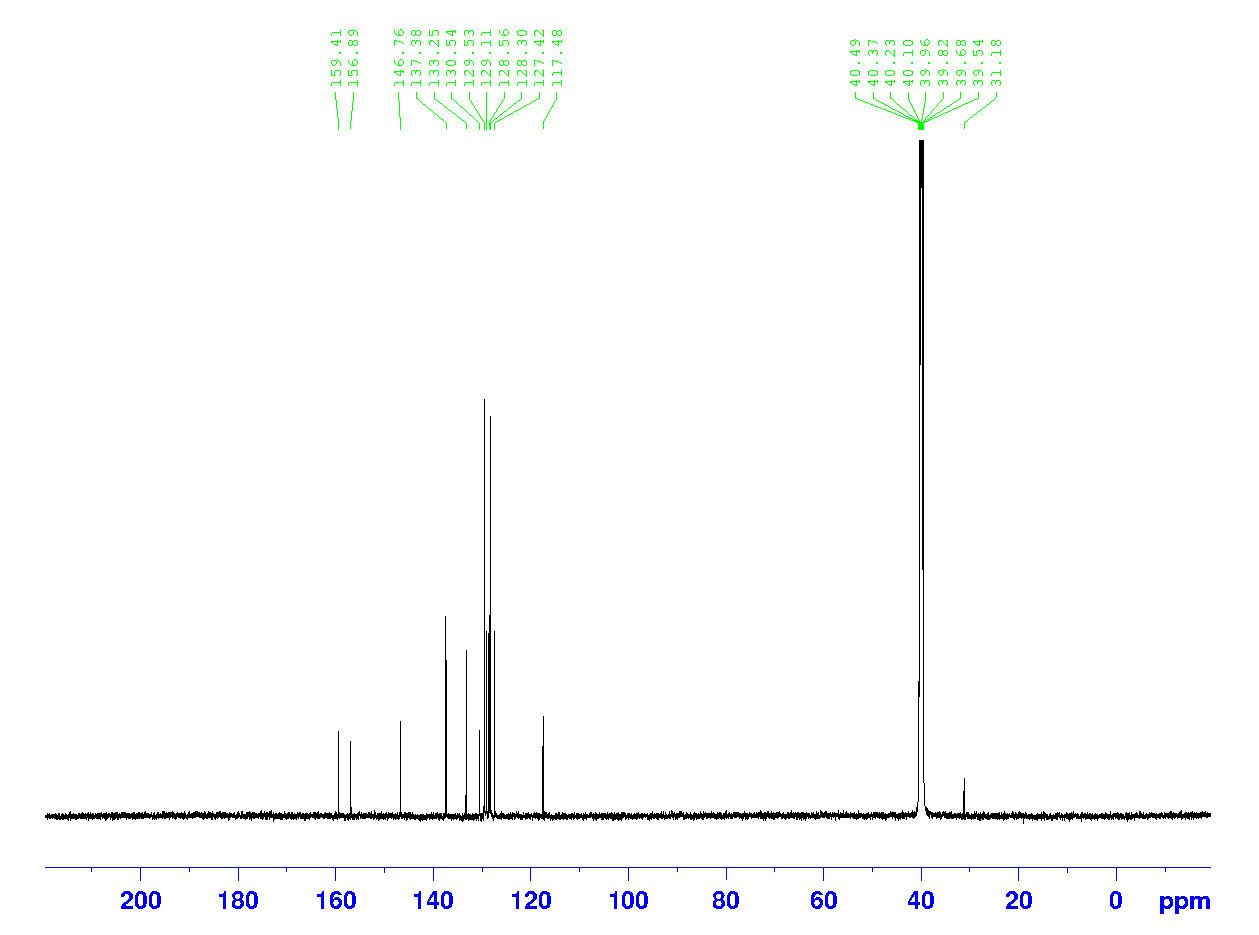


## ‎1.3 ^13^C-NMR spectrum of **2a**


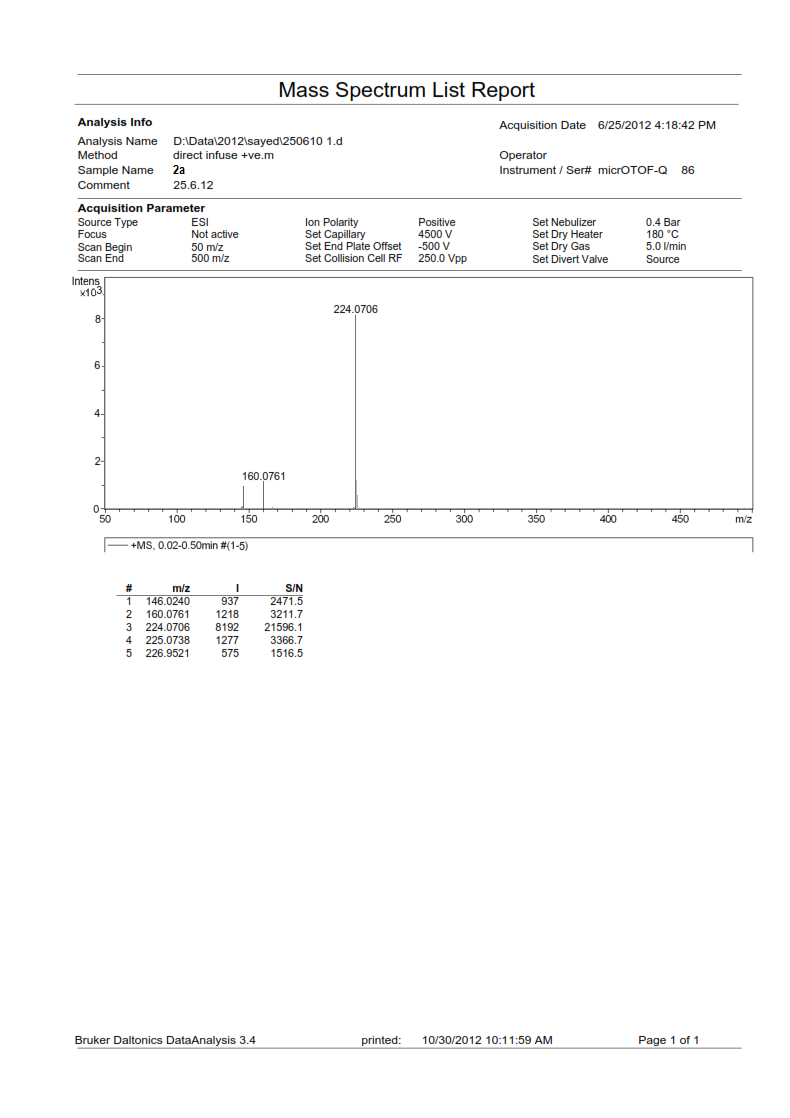


## ‎1.4 HRMS spectrum of **2a**

# The IR, 1H-NMR, 13C-NMR and HRMS spectra of product No. 2b


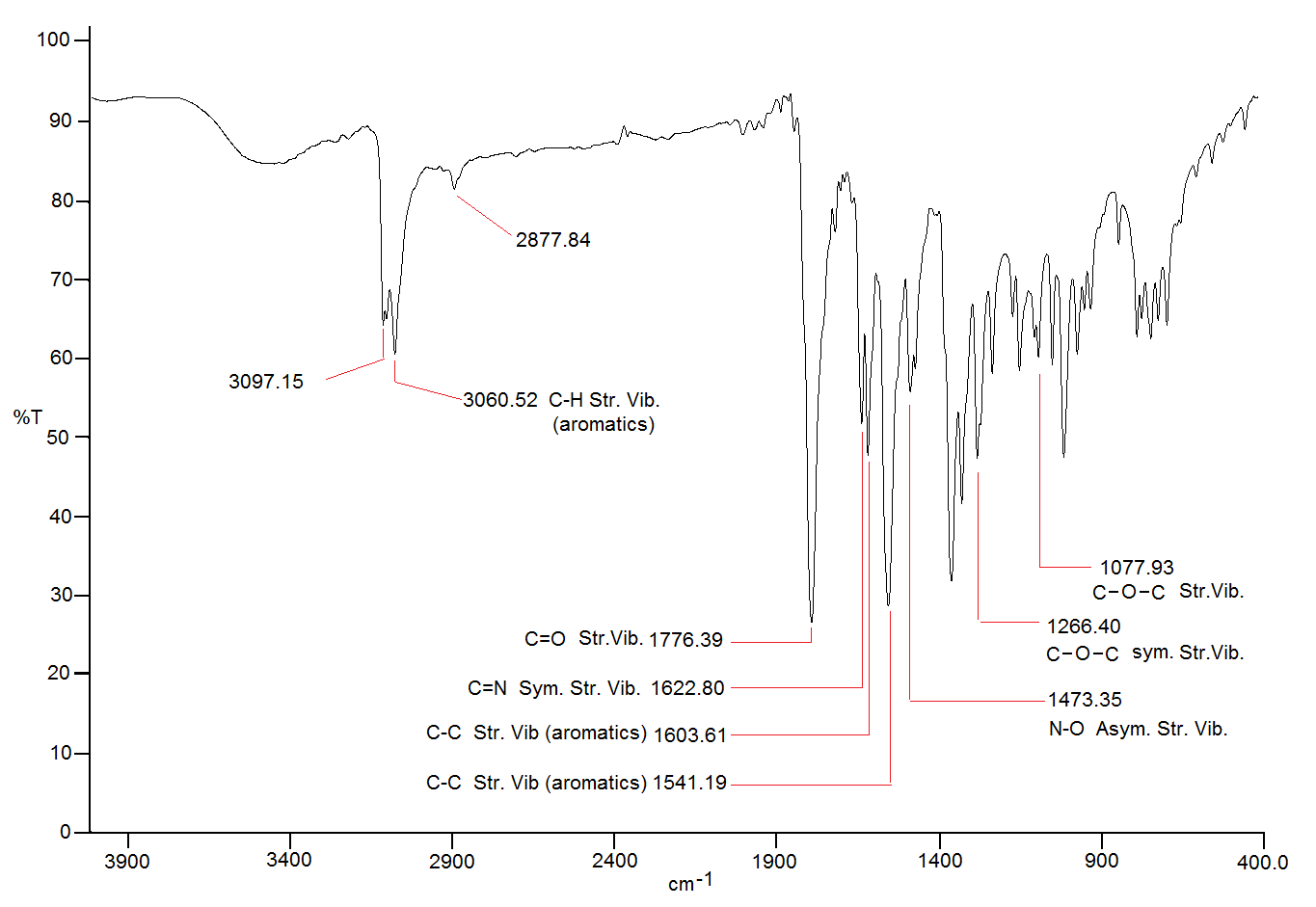


## ‎2.1 IR spectrum (KBr) of **2b**


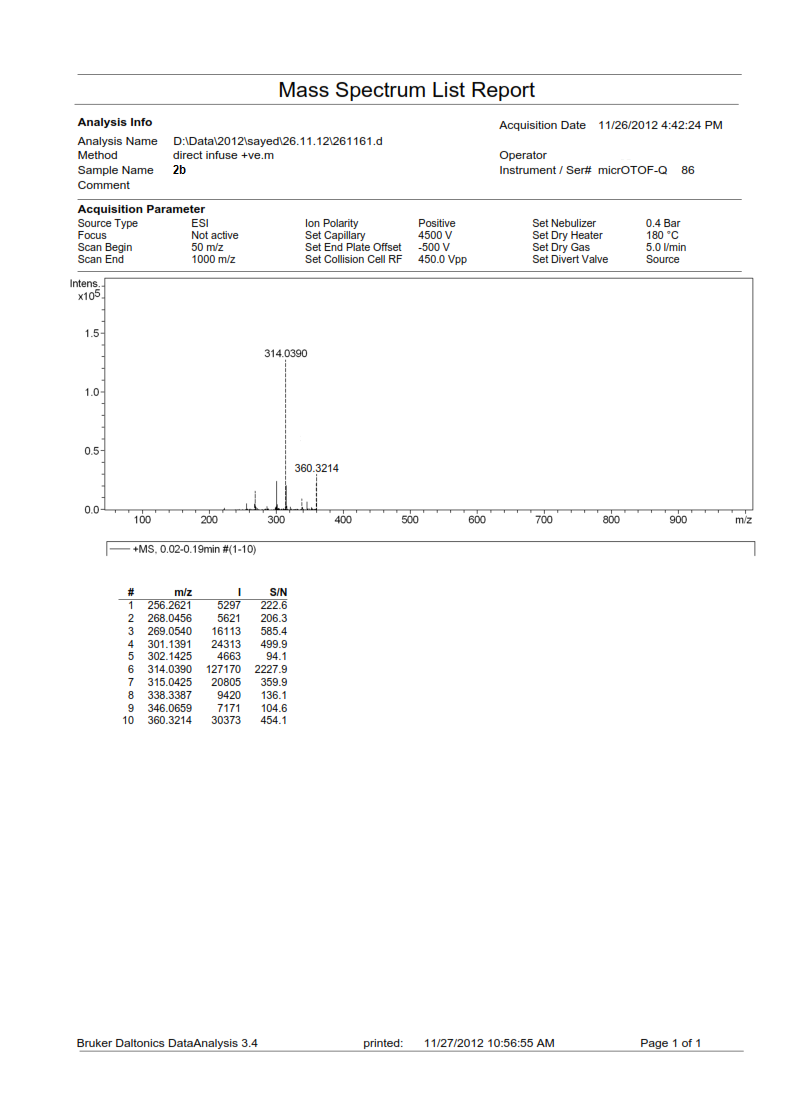


## ‎2.2 HRMS spectrum of **2b**


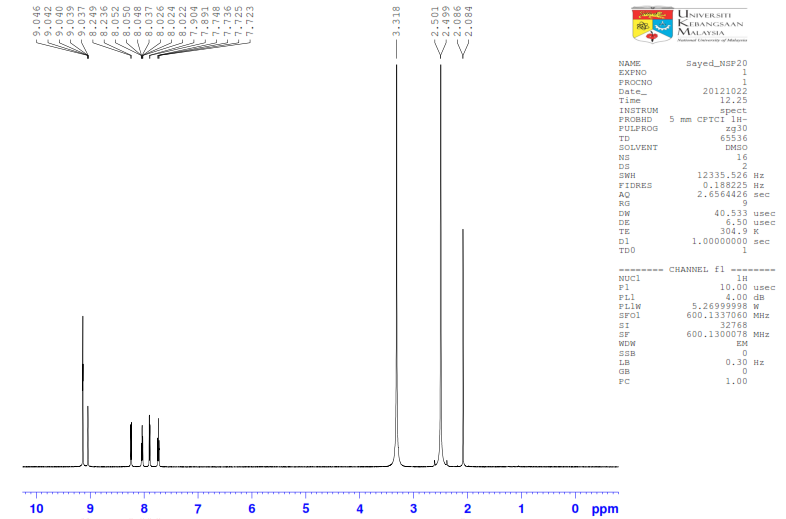


## ‎2.3 ^1^H-NMR spectrum of **2b**


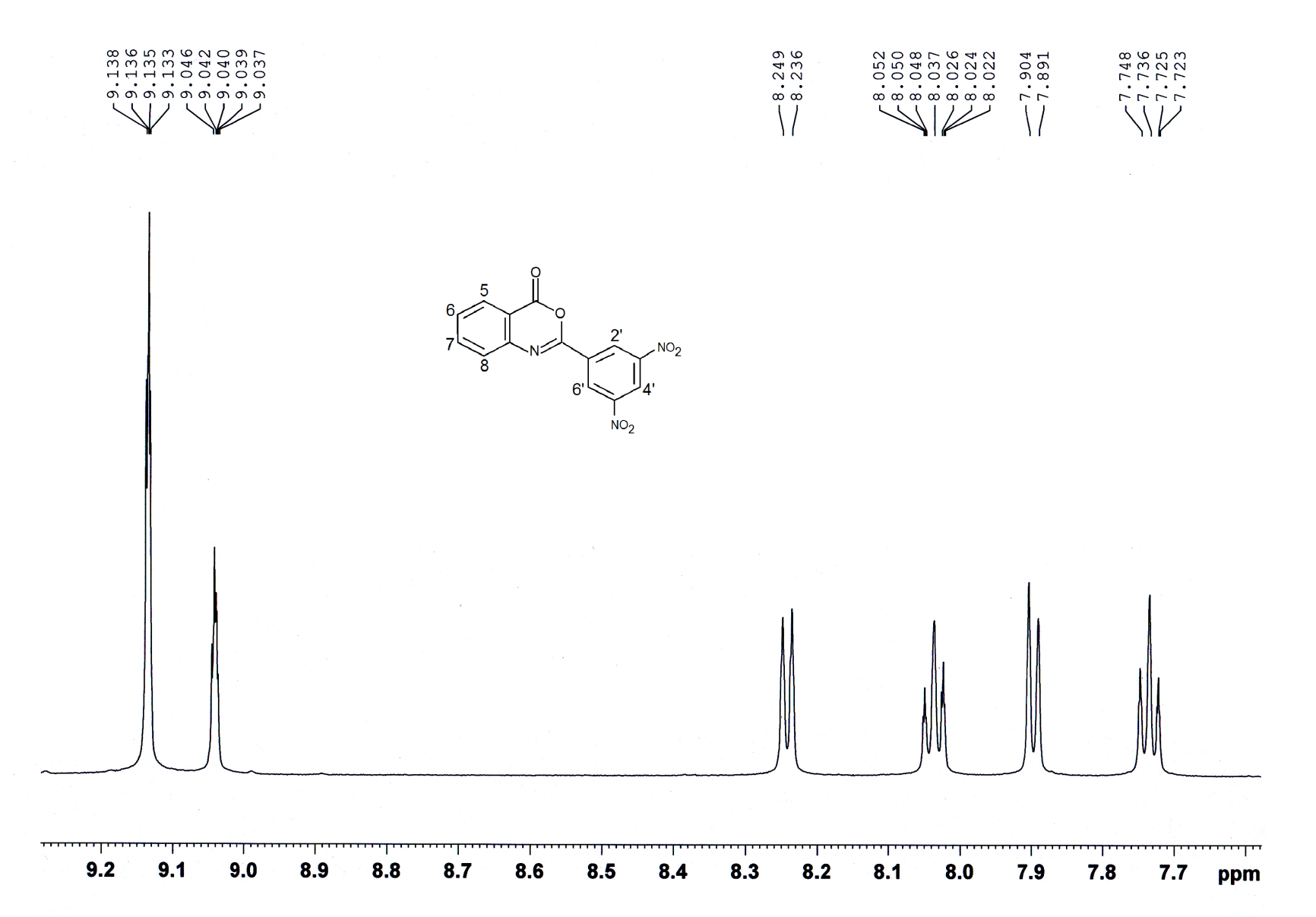


## ‎2.4 Enlarged ^1^H-NMR spectrum of **2b**


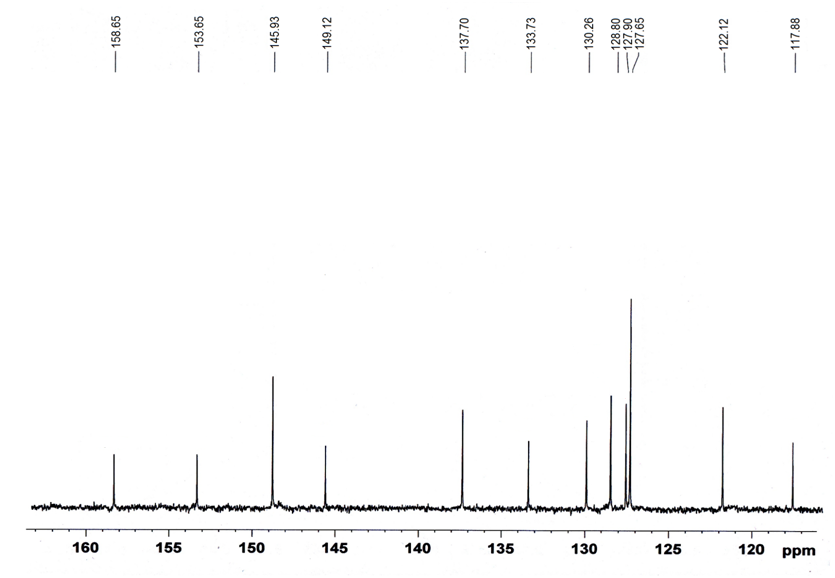


## ‎2.5 ^13^C-NMR spectrum of **2b**

# The IR, 1H-NMR, 13C-NMR and HRMS spectra of product No. 2c


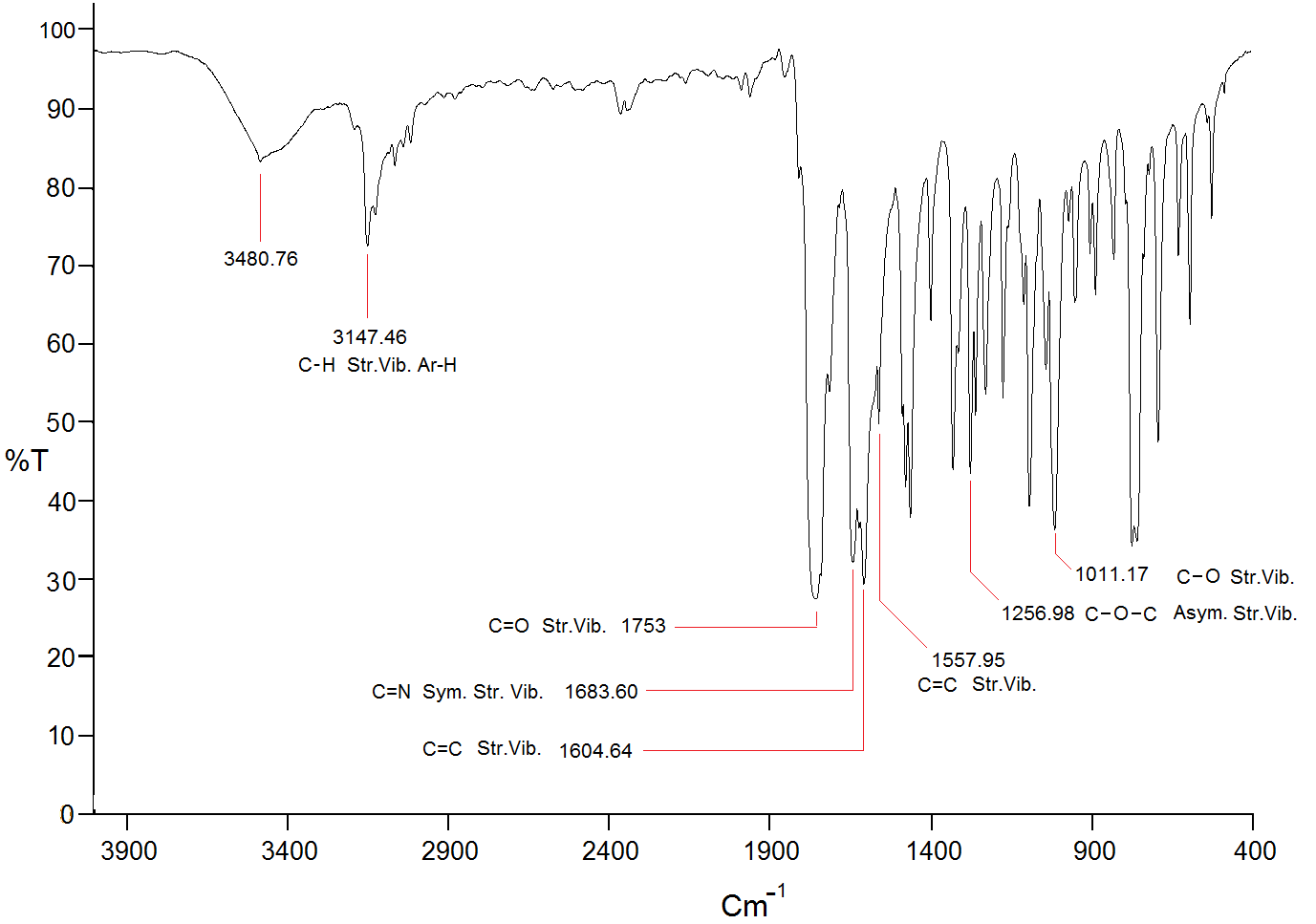


## ‎3.1 IR spectrum of **2c**


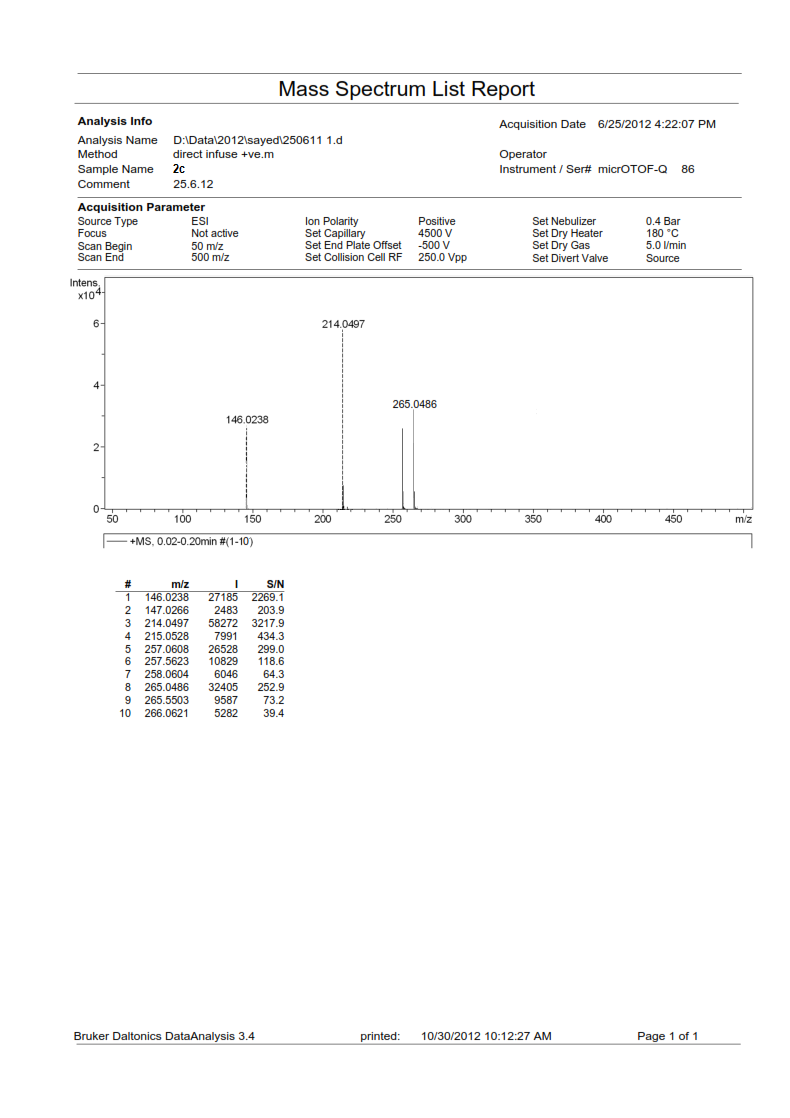


## ‎3.2 HRMS spectrum of **2c**


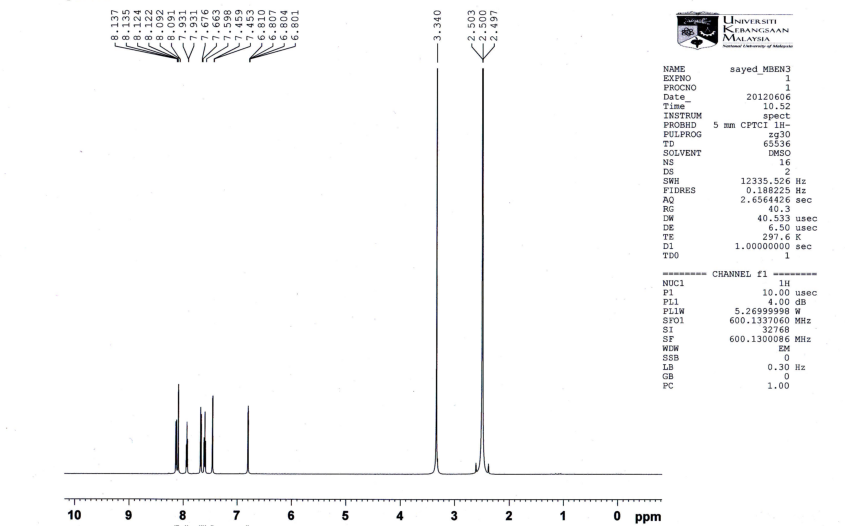


## ‎3.3 ^1^H-NMR spectrum of **2c**


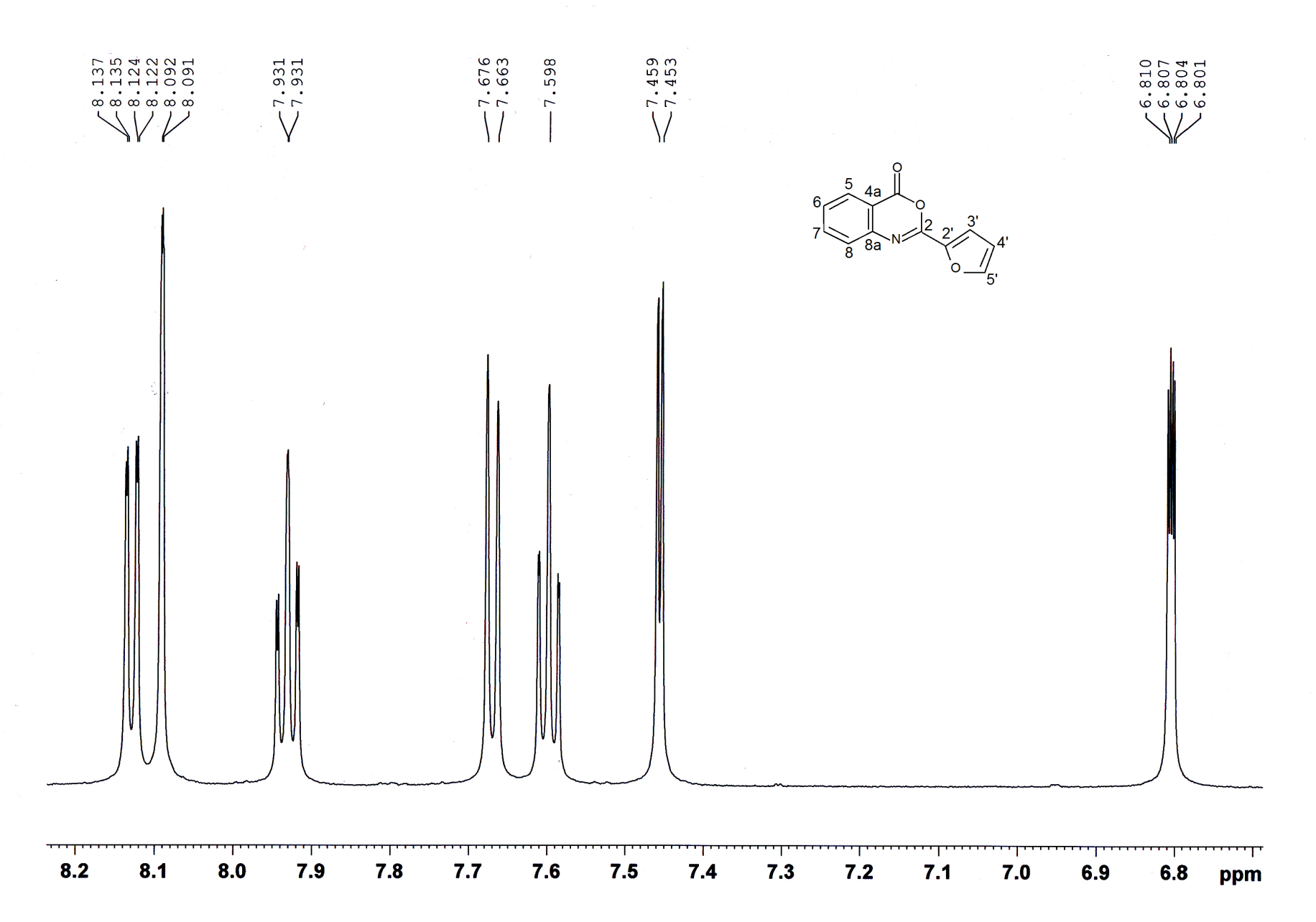


## ‎3.4 Enlarged ^1^H-NMR spectrum of **2c**


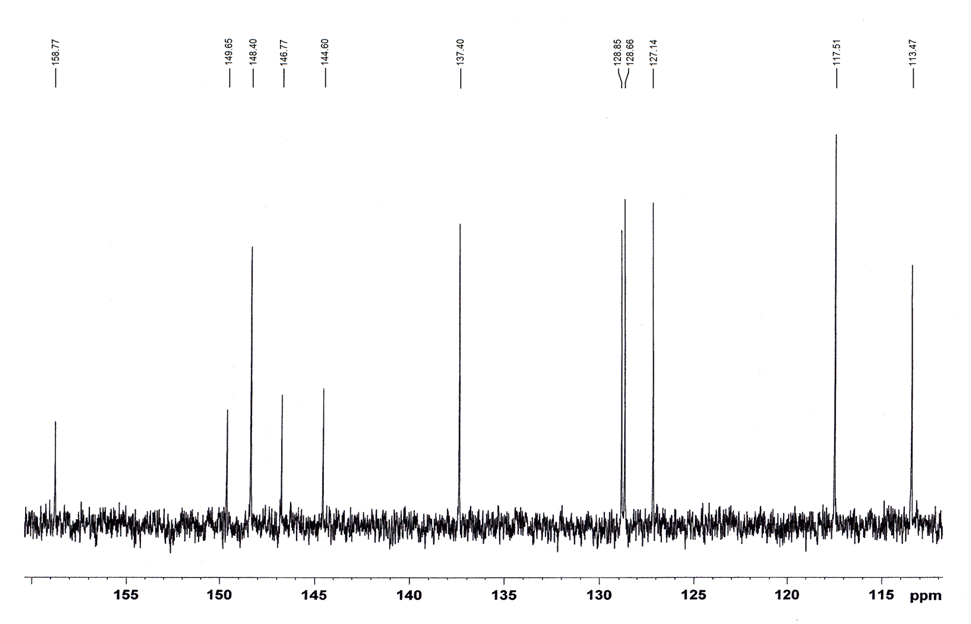


## ‎3.5 ^13^C-NMR spectrum of **2c**

# The IR, 1H-NMR, 13C-NMR and HRMS spectra of product No. 2d


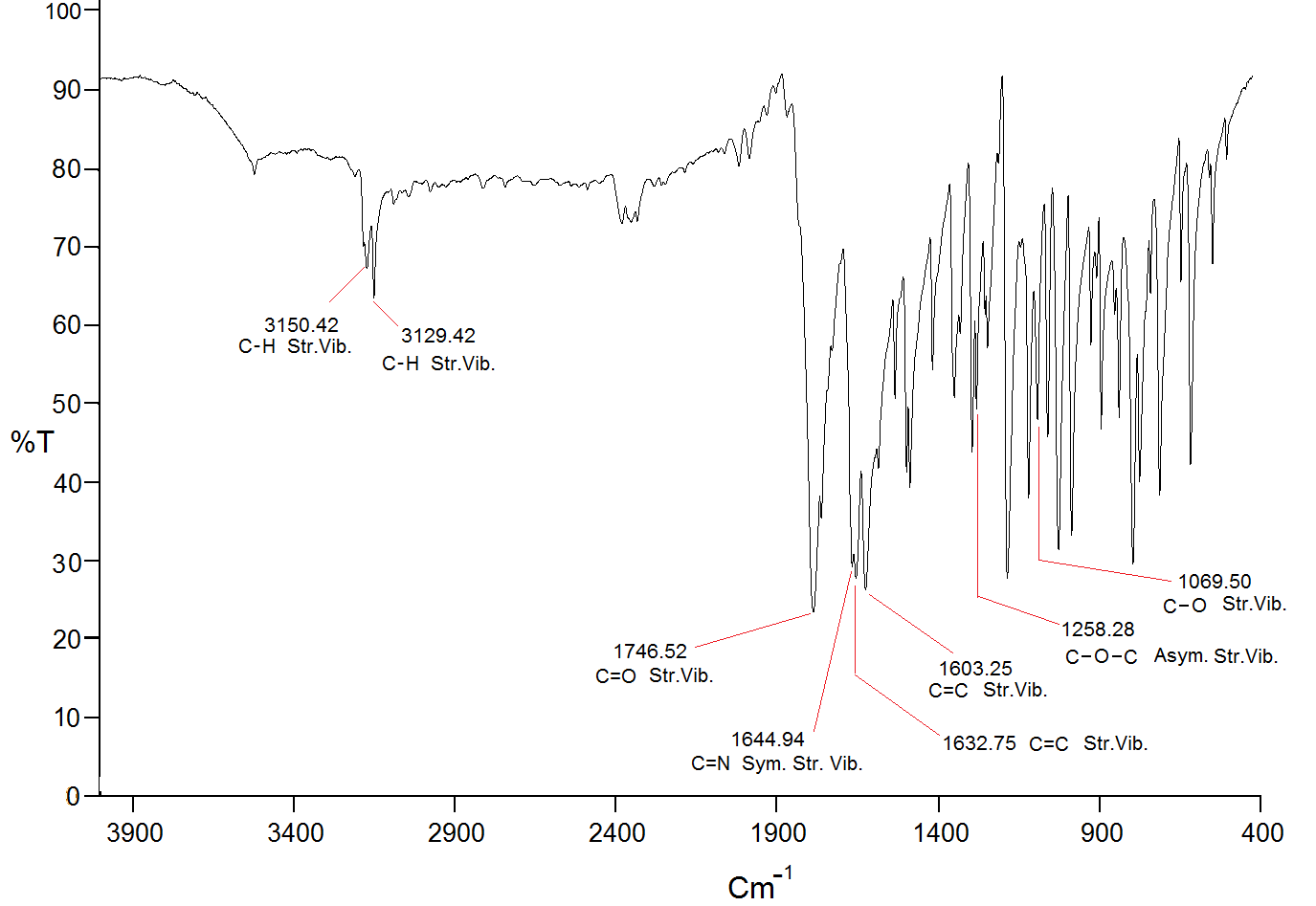


## ‎4.1 IR spectrum of **2d**


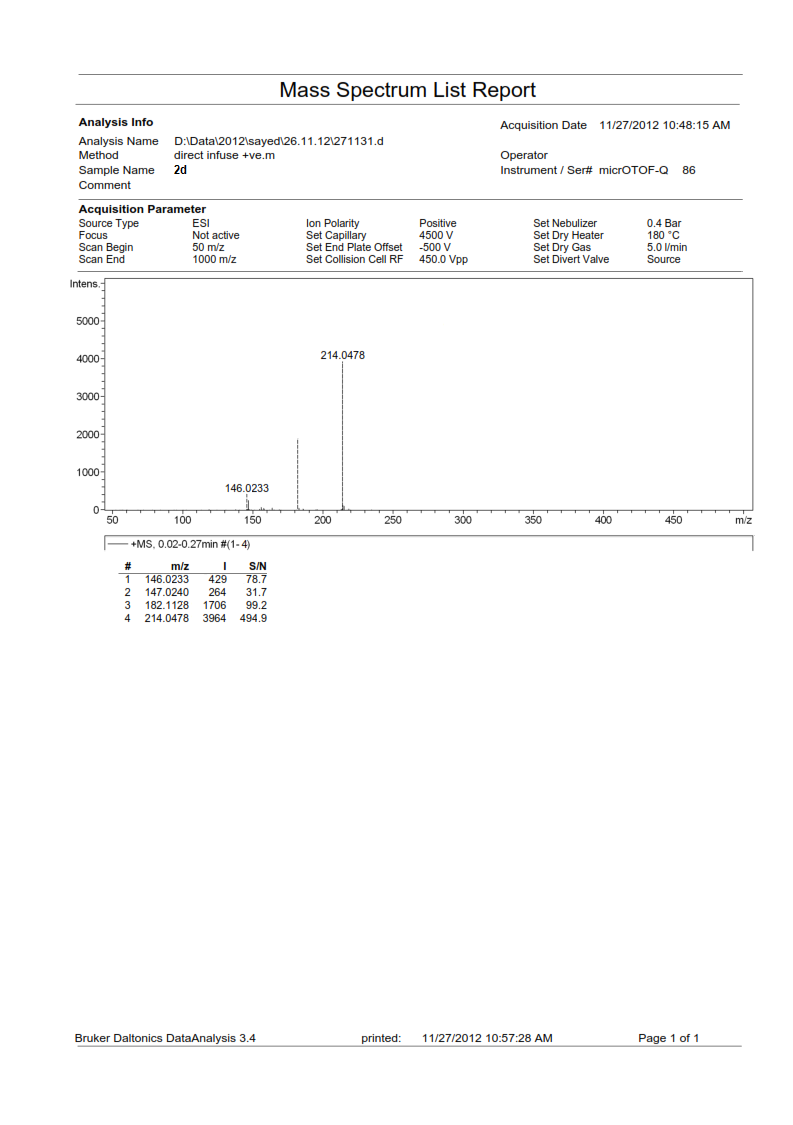


## ‎4.2 HRMS spectrum of **2d**


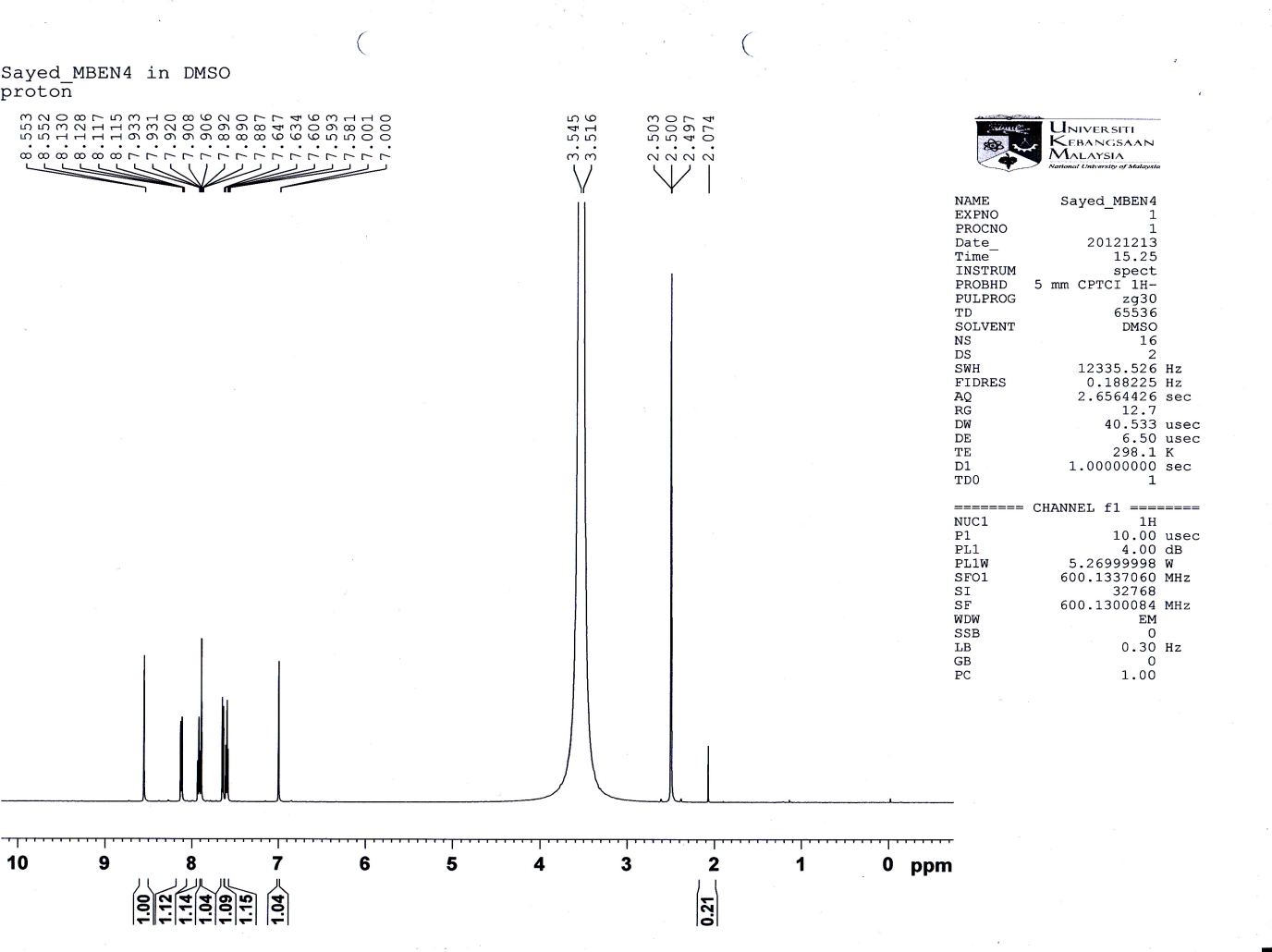


## ‎4.3 ^1^H-NMR spectrum of **2d**


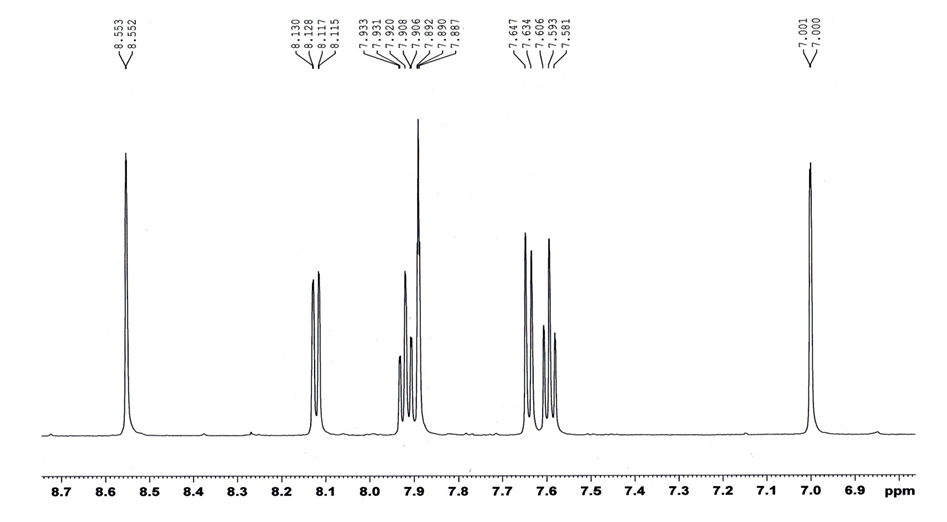


## ‎4.4 Enlarged ^1^H-NMR spectrum of **2d**

^
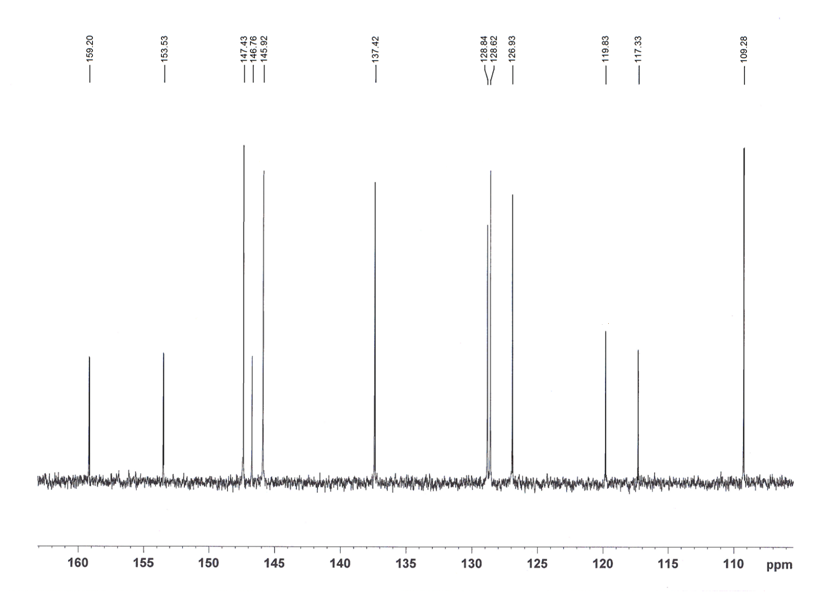
^

## ‎4.5 ^13^C-NMR spectrum of **2d**

# The IR, 1H-NMR, 13C-NMR and HRMS spectra of product No. 2e


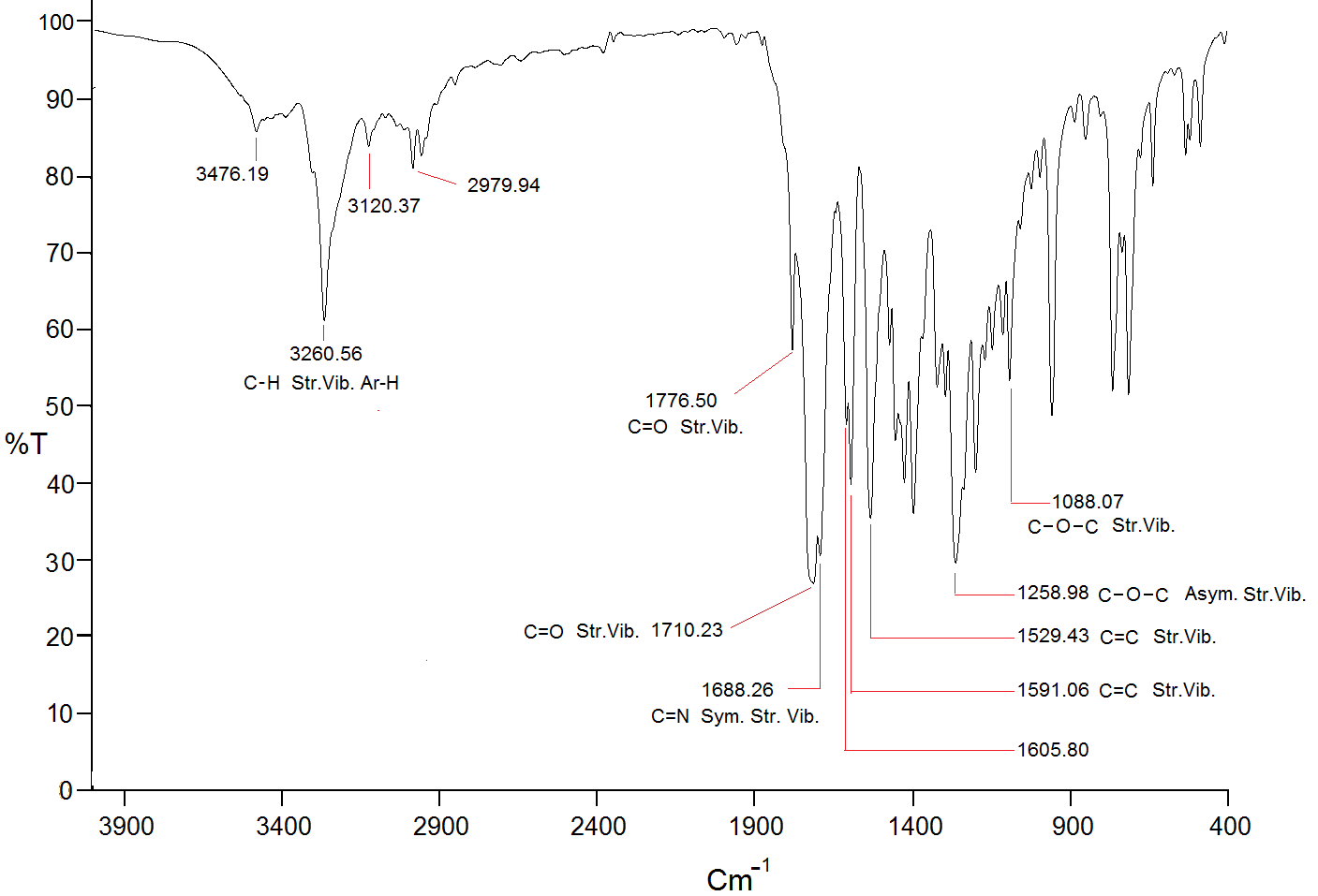


## ‎5.1 IR spectrum of **2e**


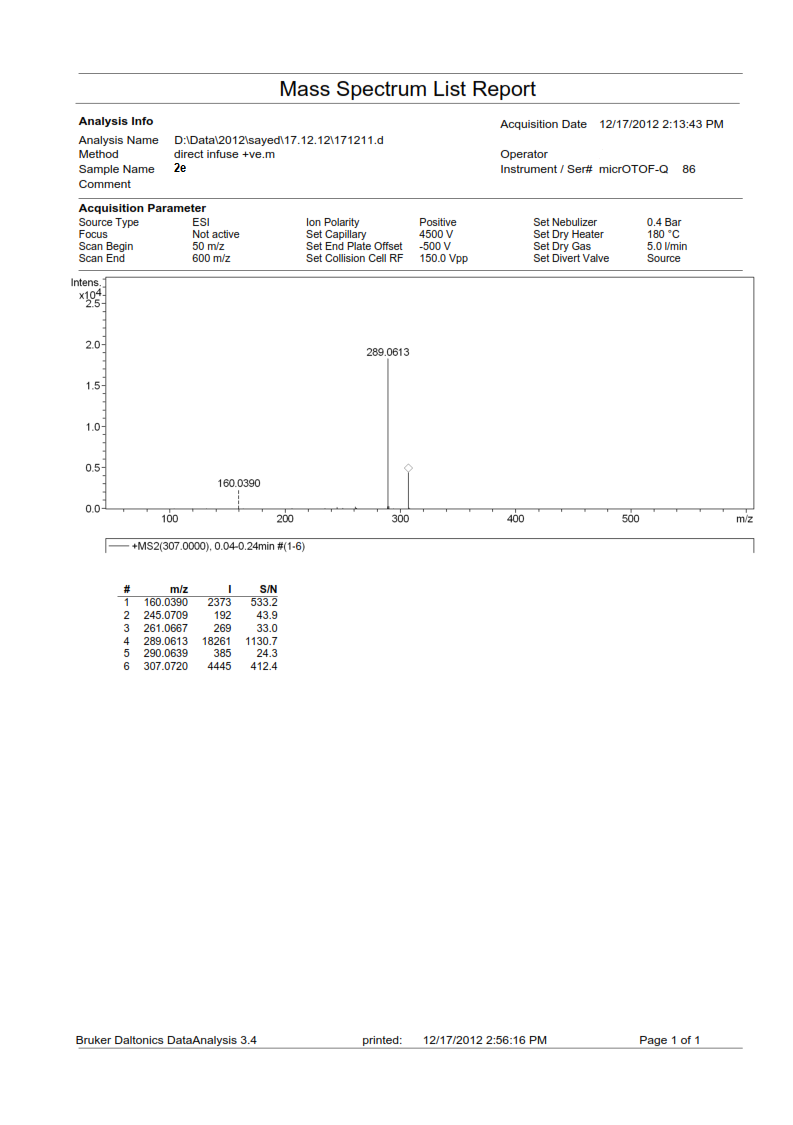


## ‎5.2 HRMS spectrum of **2e**


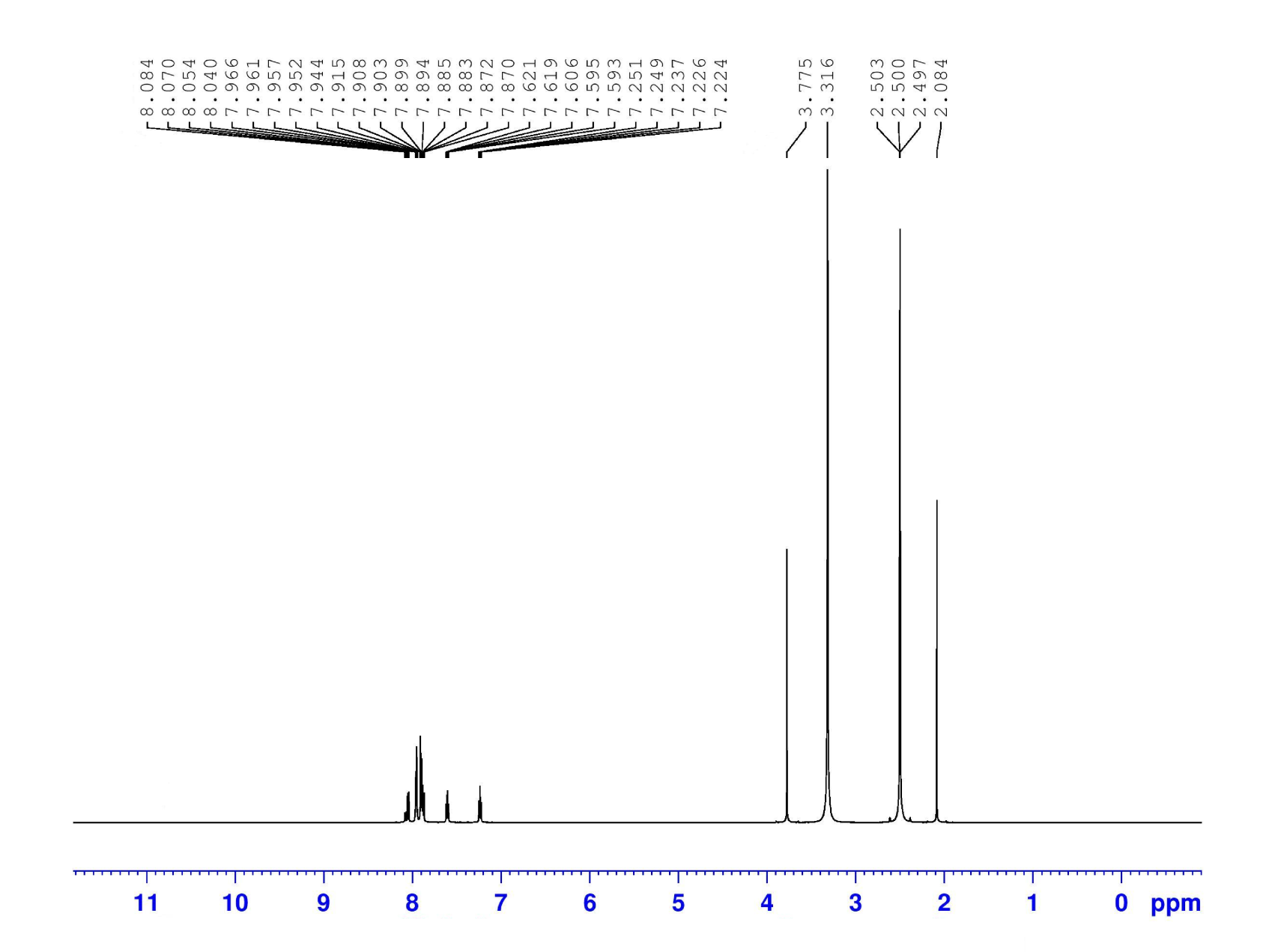


## ‎5.3 ^1^H-NMR spectrum of **2e**


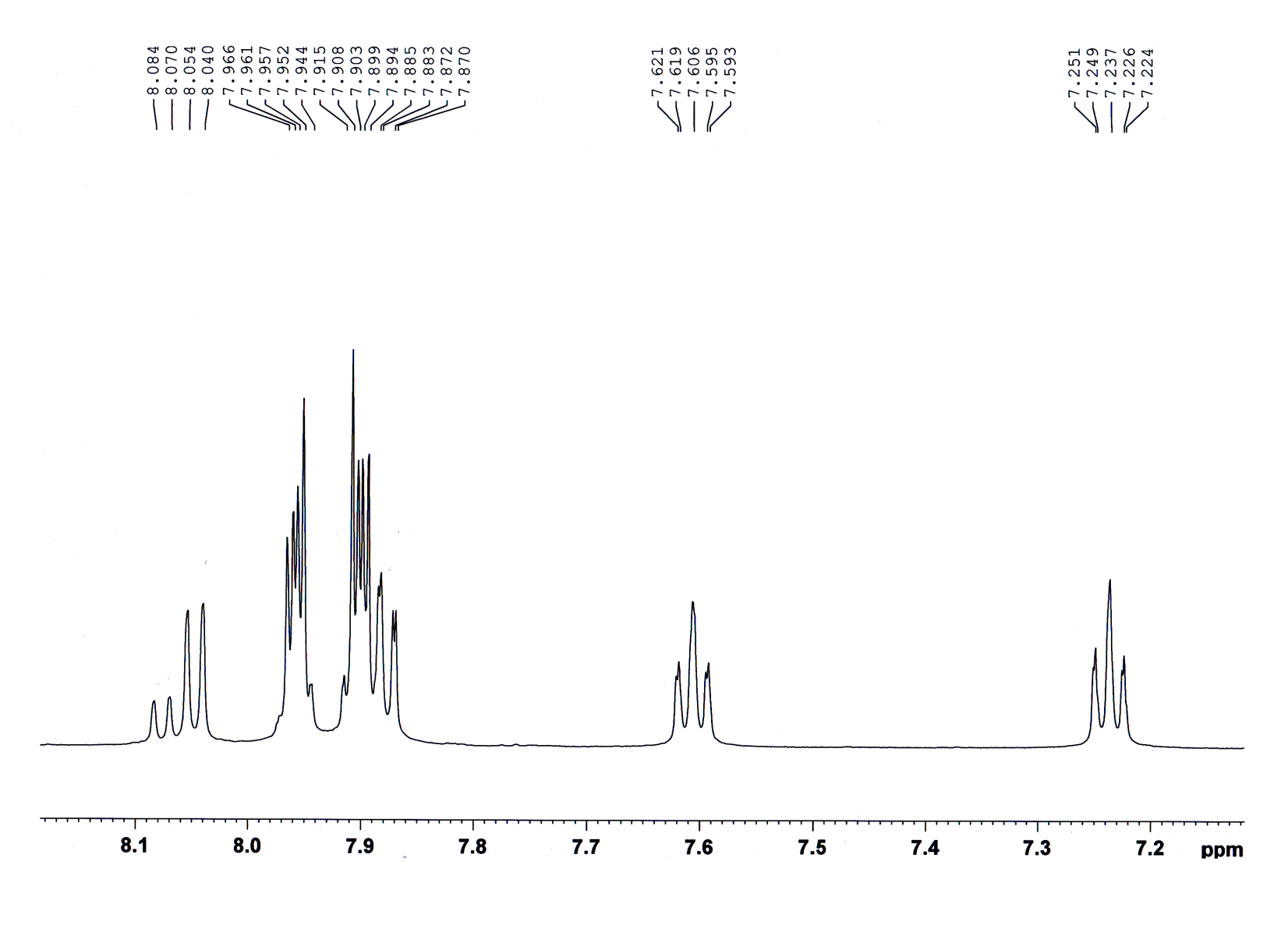


## ‎5.4 Enlarged H-NMR spectrum of **2e**


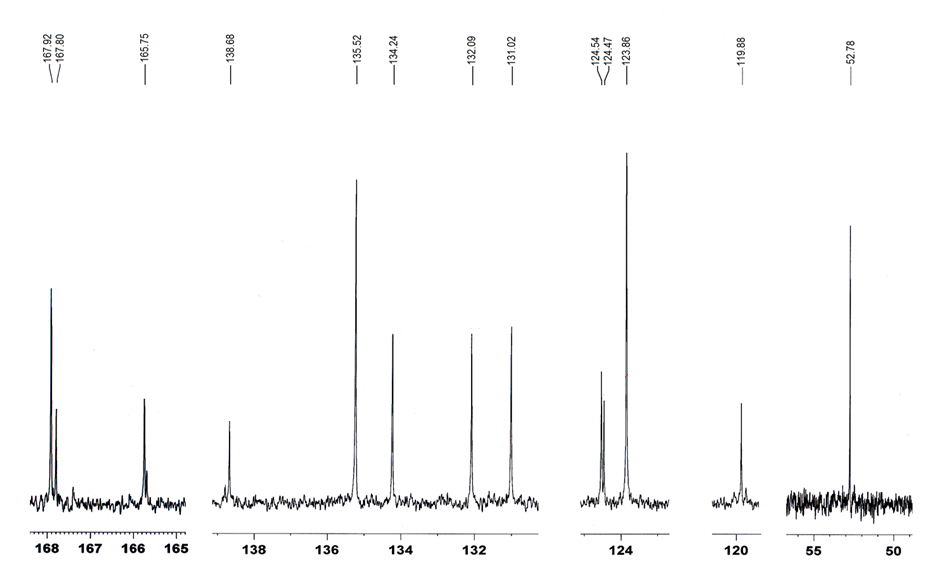


## ‎5.5 Enlarged ^13^C-NMR spectrum of **2e**

# The IR, 1H-NMR, 13C-NMR and HRMS spectra of product No. 2f


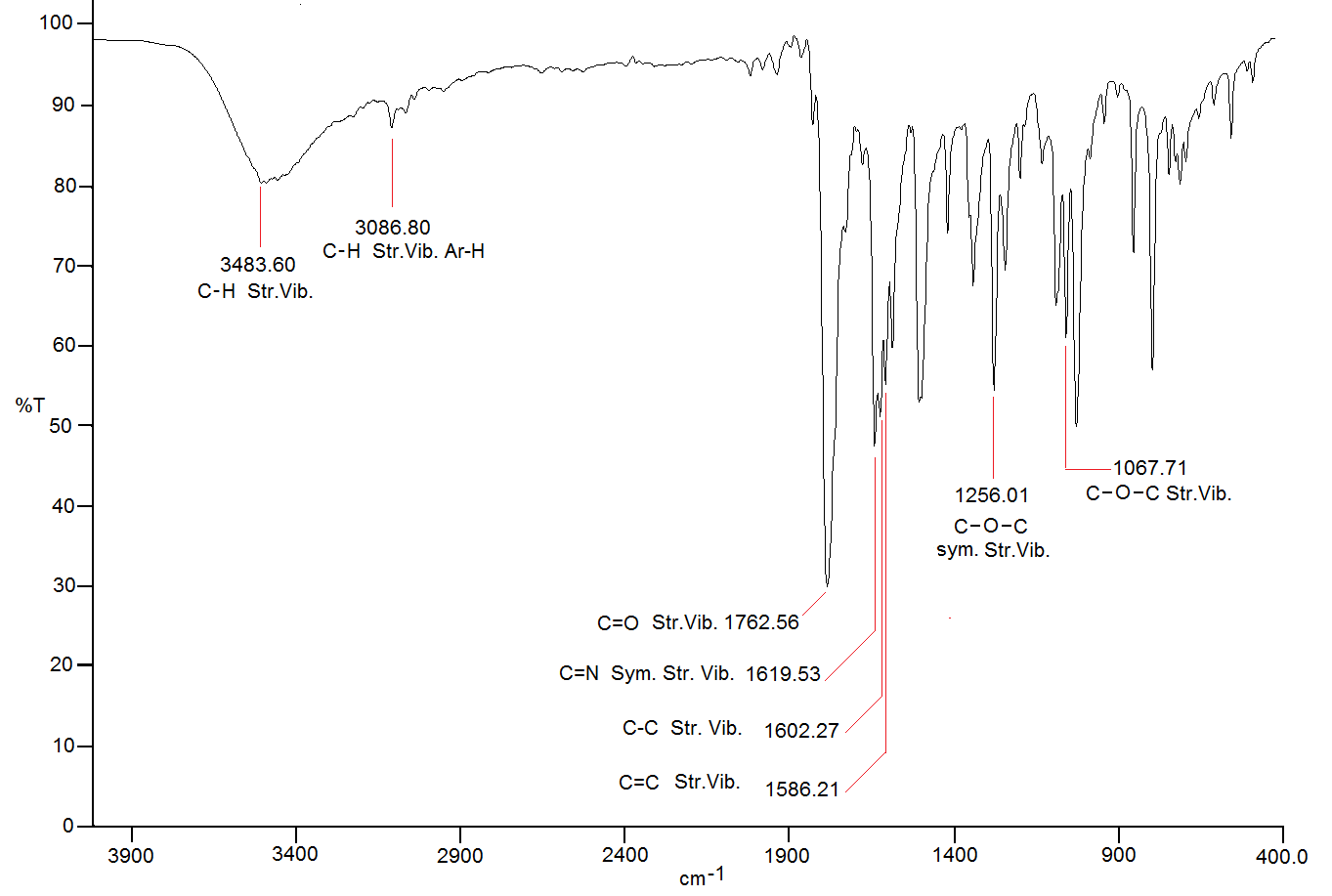


## ‎6.1 FT-IR spectrum of **2f**


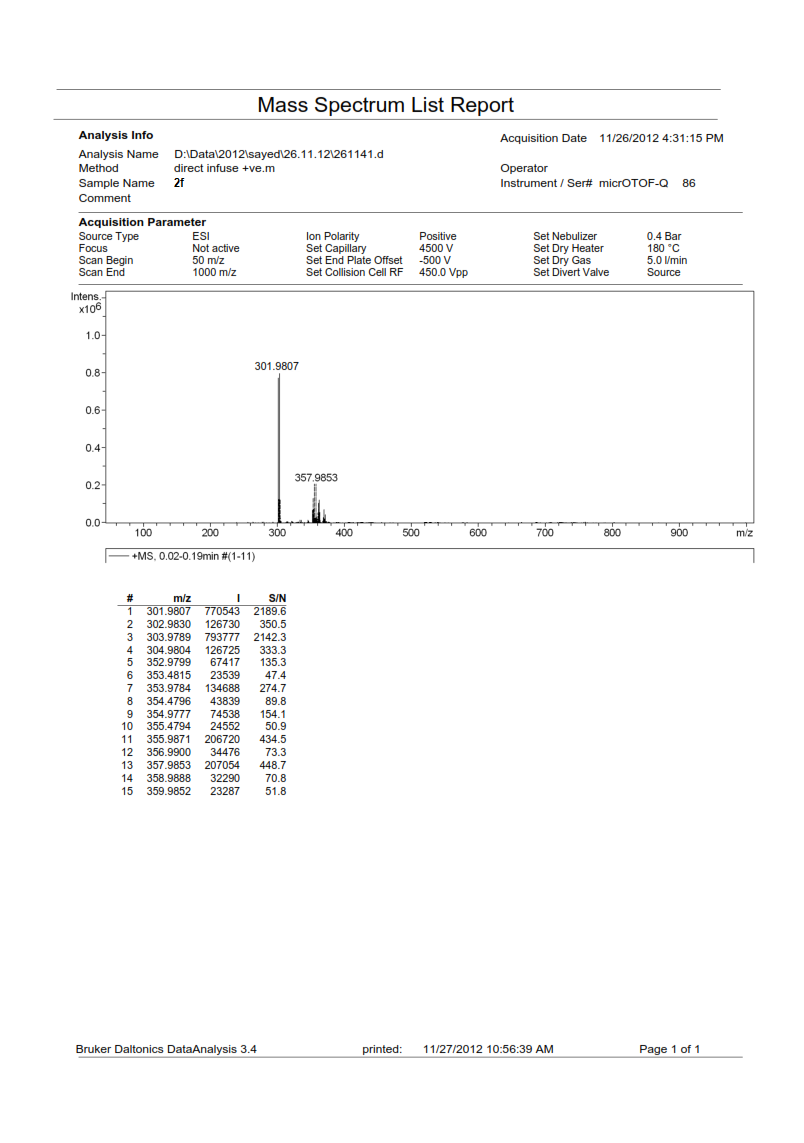


## ‎6.2 HRMS spectrum of **2f**


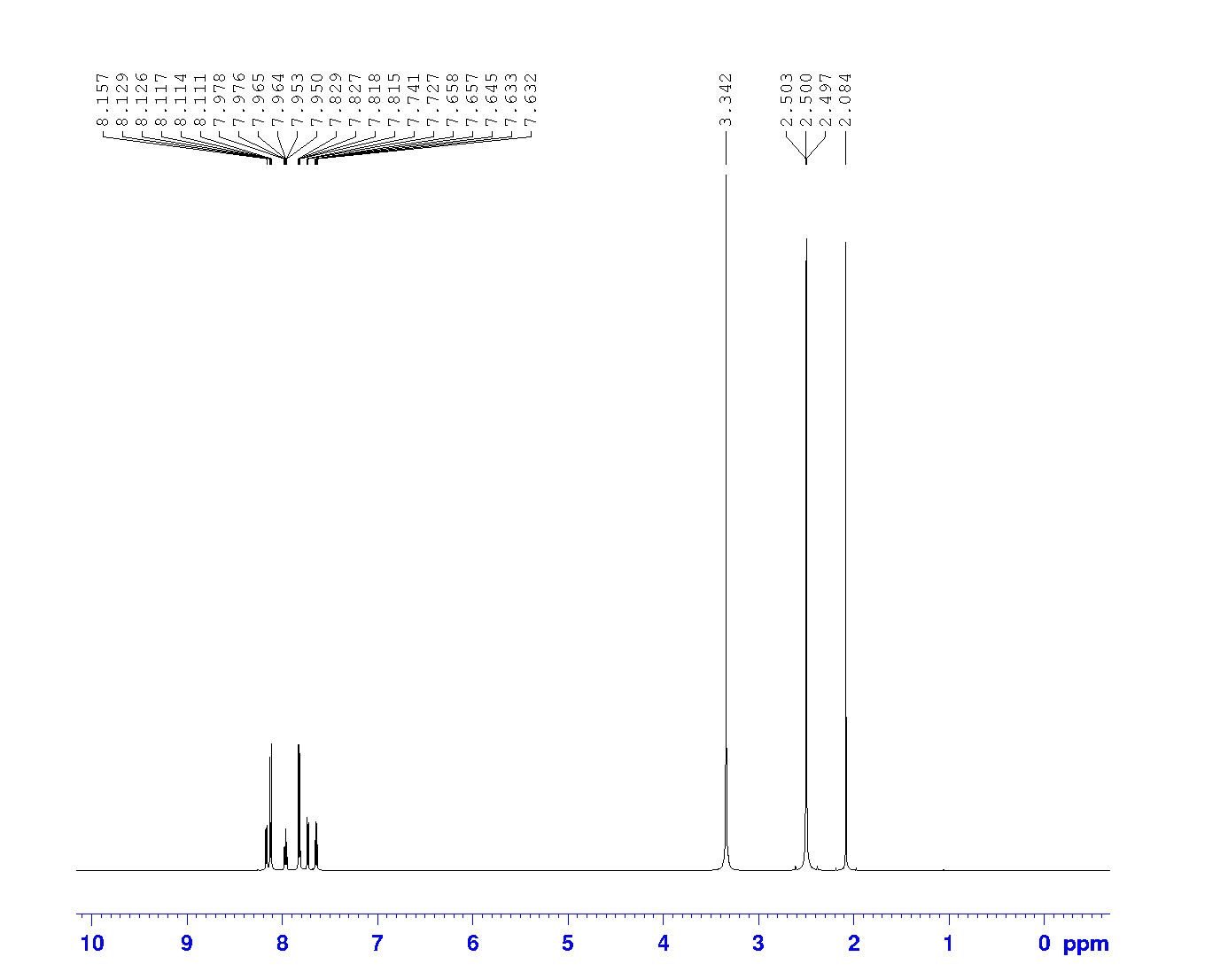


## ‎6.3 ^1^H-NMR of **2f**


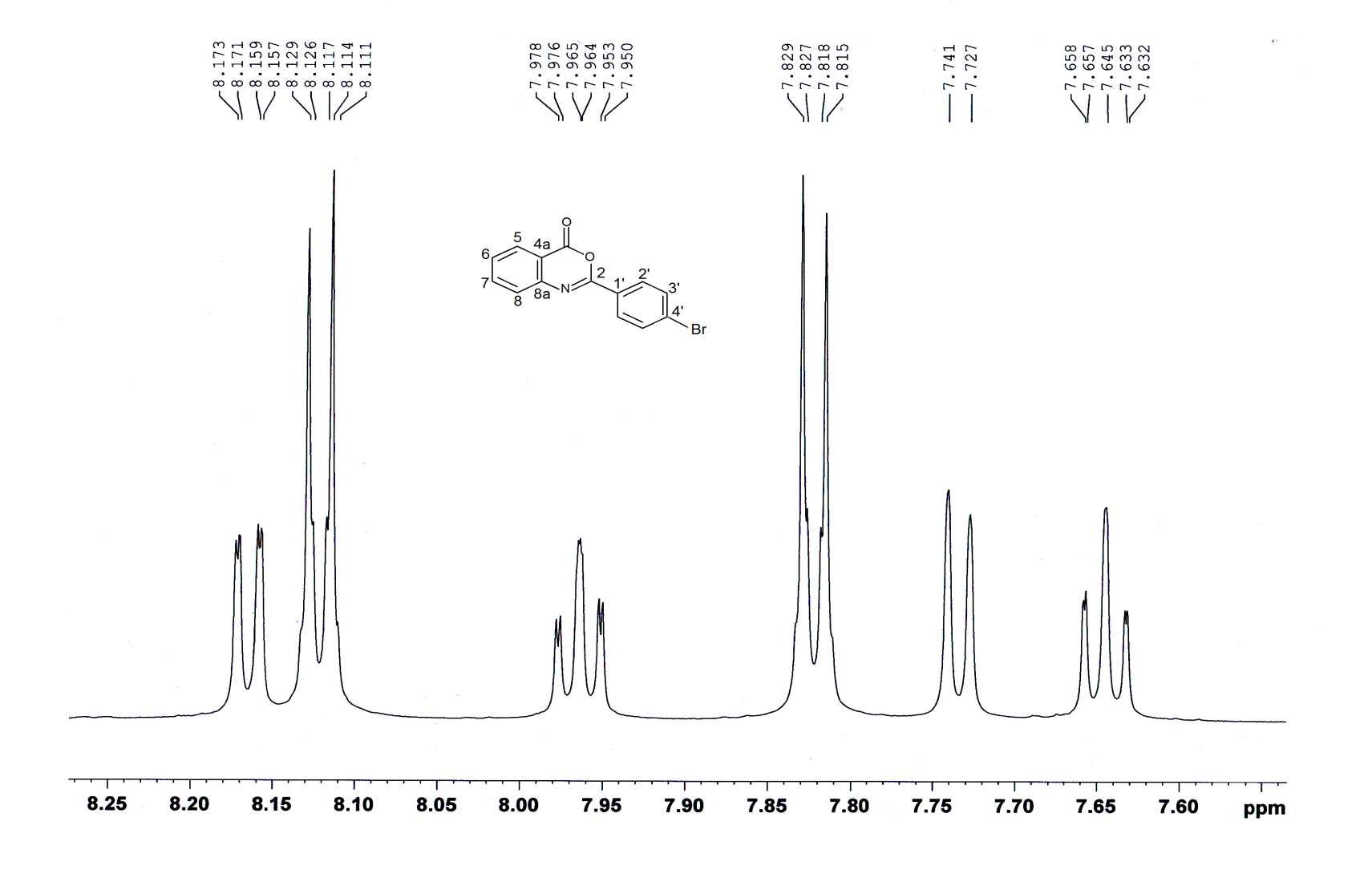


## ‎6.4 Enlarged ^1^H-NMR of **2f**


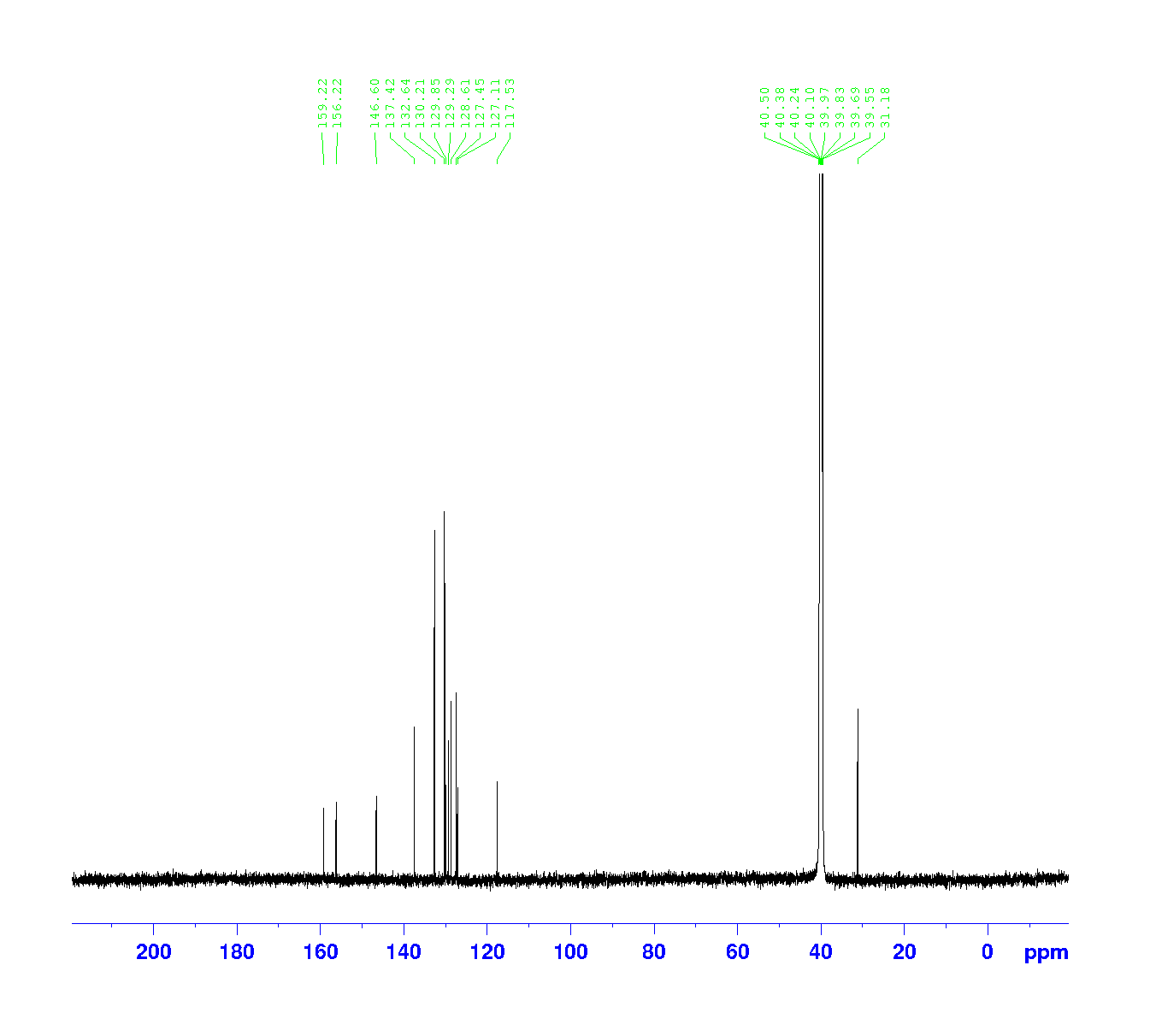


## ‎6.5 ^13^C-NMR of **2f**


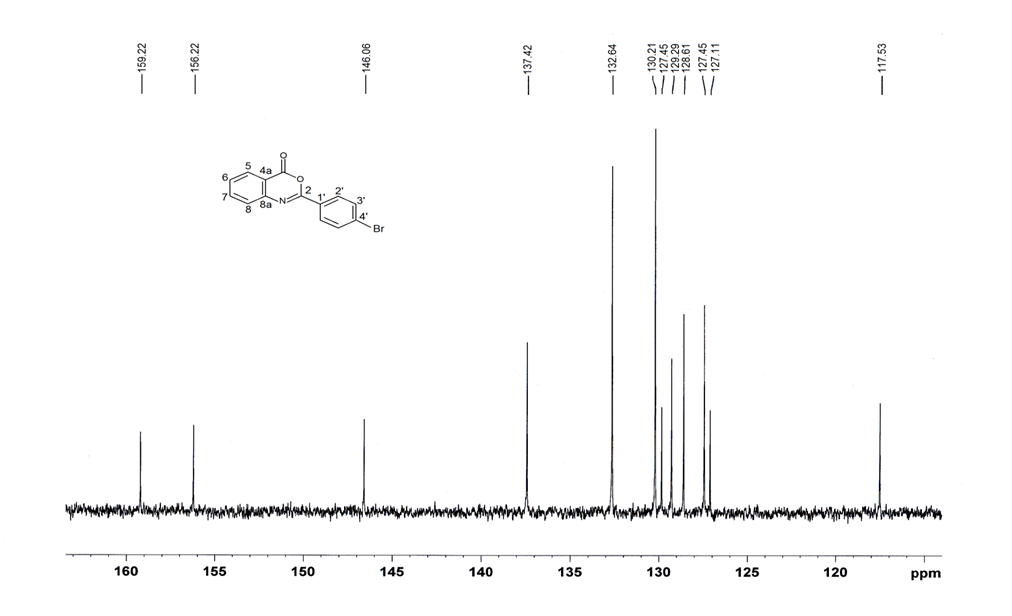


## ‎6.6 Enlarged ^13^C-NMR of **2f**

# The IR, 1H-NMR, 13C-NMR and HRMS spectra of product No. 2g


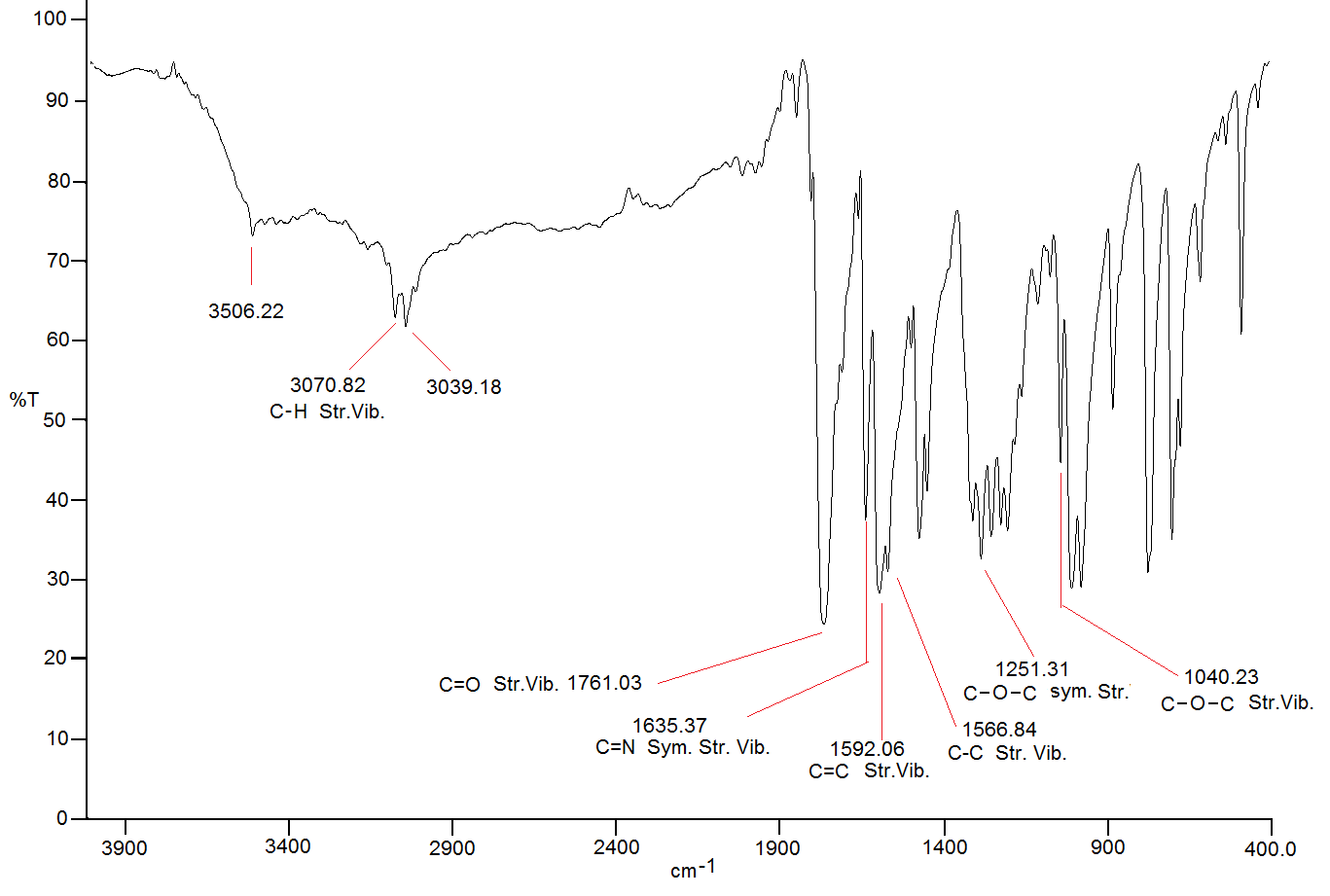


## ‎7.1 IR spectrum of **2g**


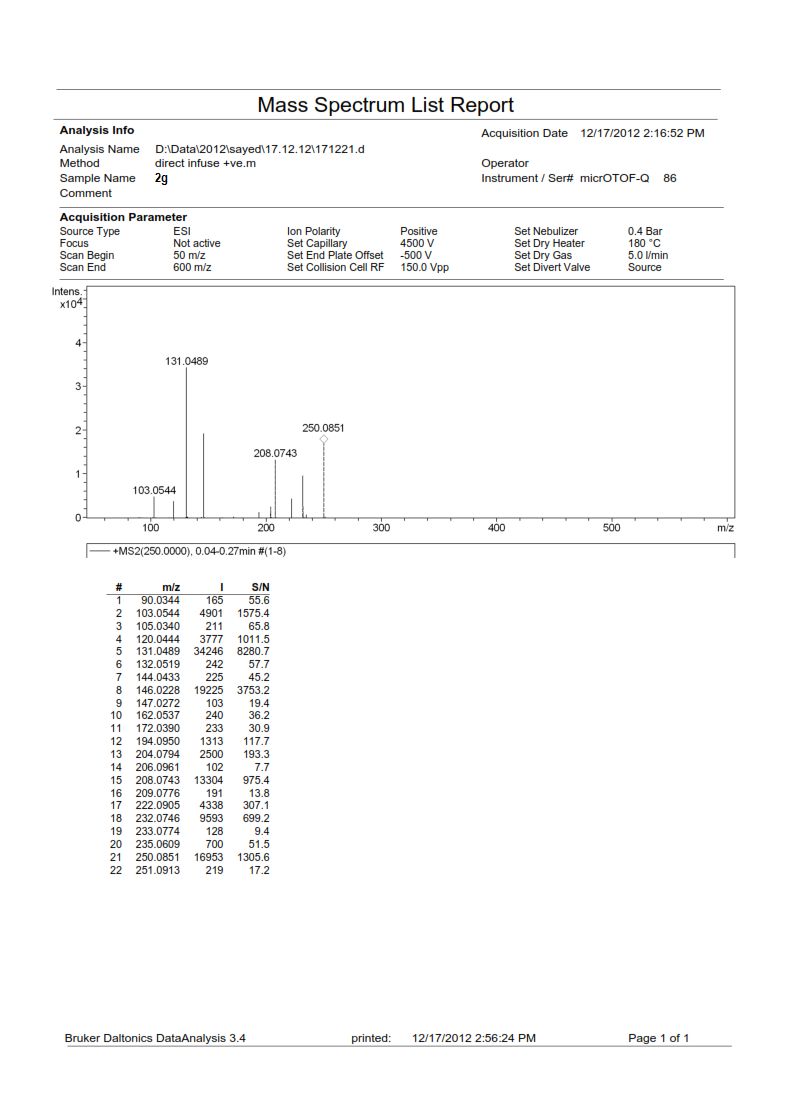


## ‎7.2 HRMS (MS-MS) of **2g**


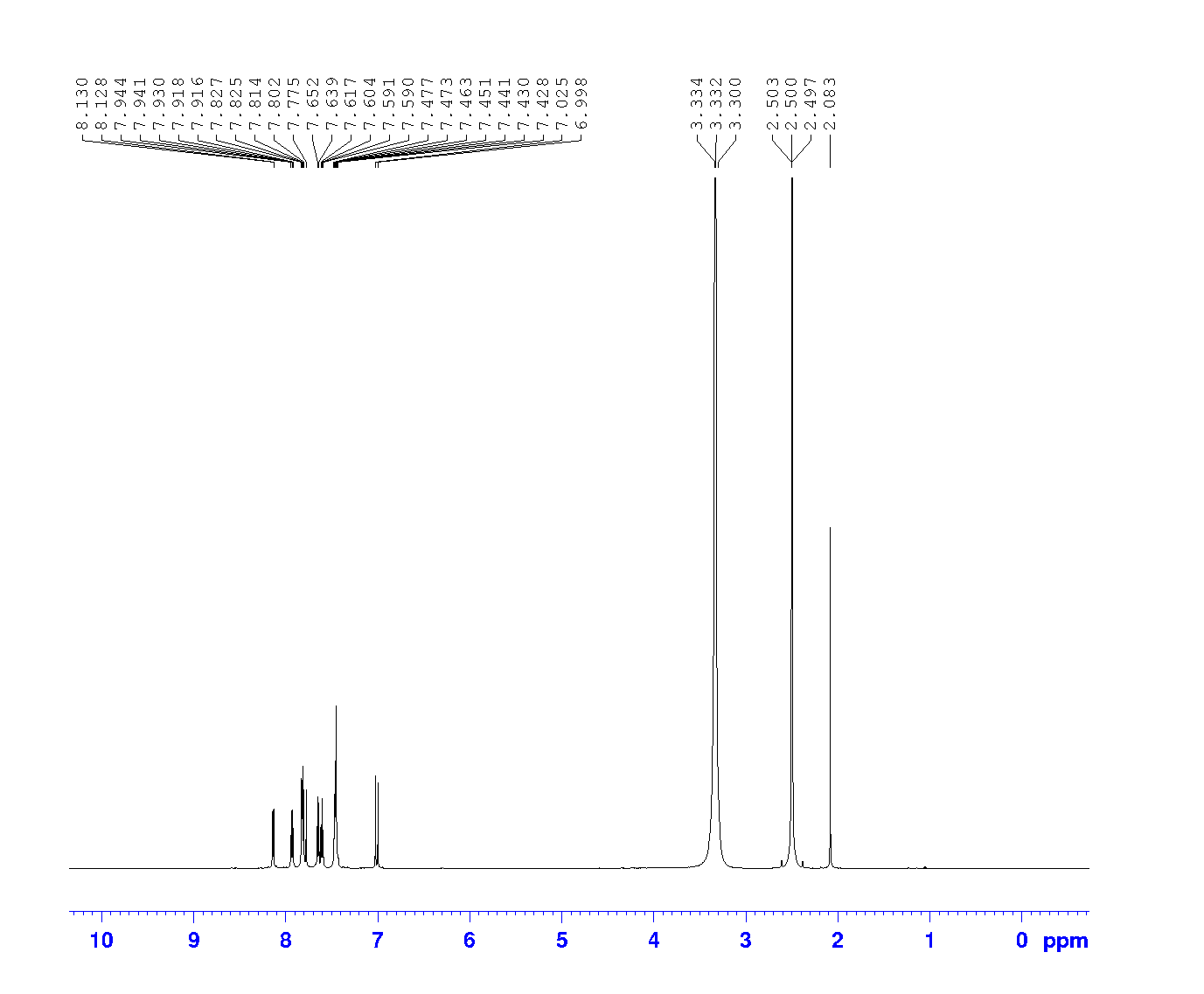


## ‎7.3 ^1^H-NMR spectrum of **2g**


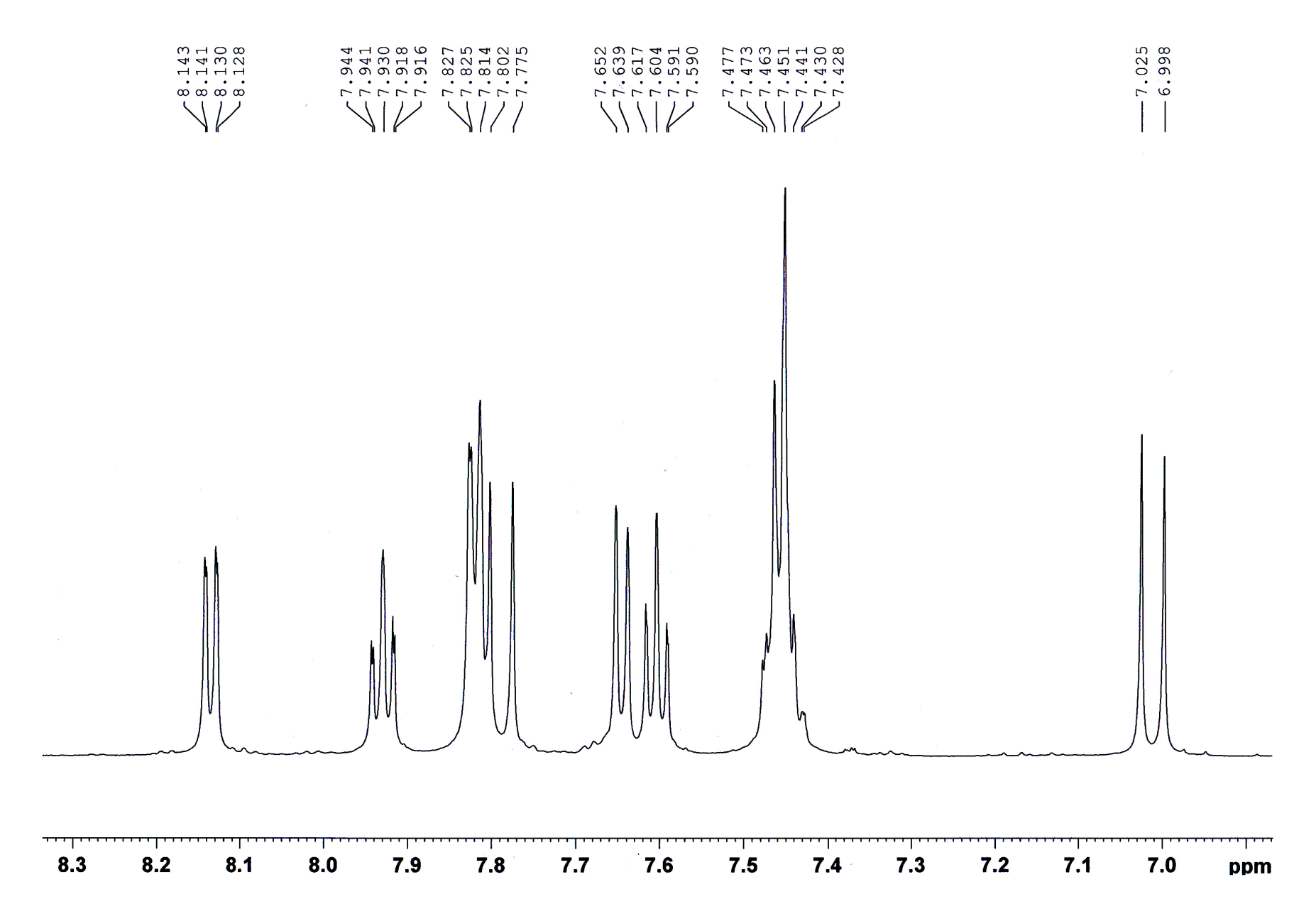


## ‎7.4 Enlarged ^1^H-NMR spectrum of **2g**


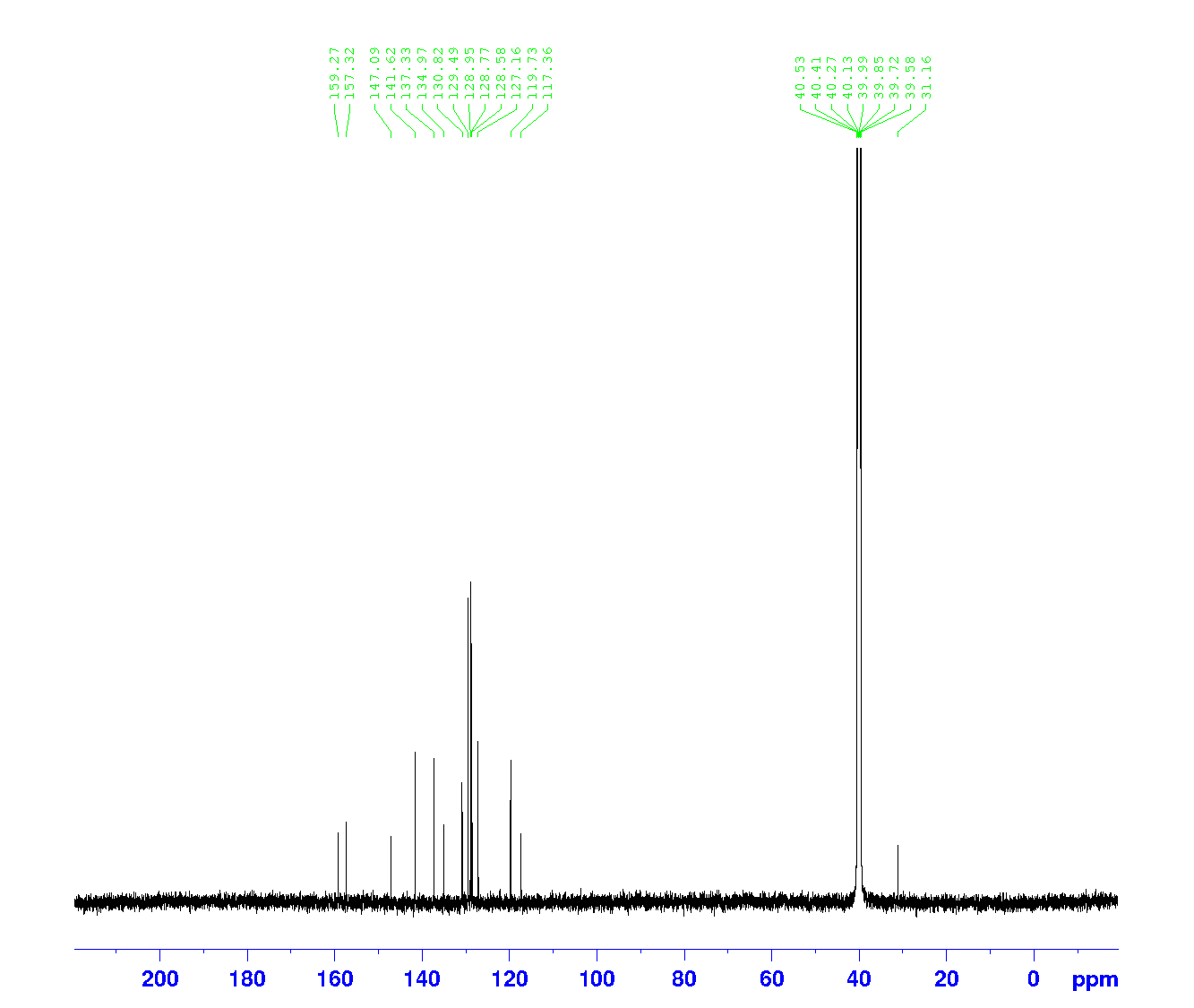


## ‎7.5 ^13^C-NMR spectrum of **2g**


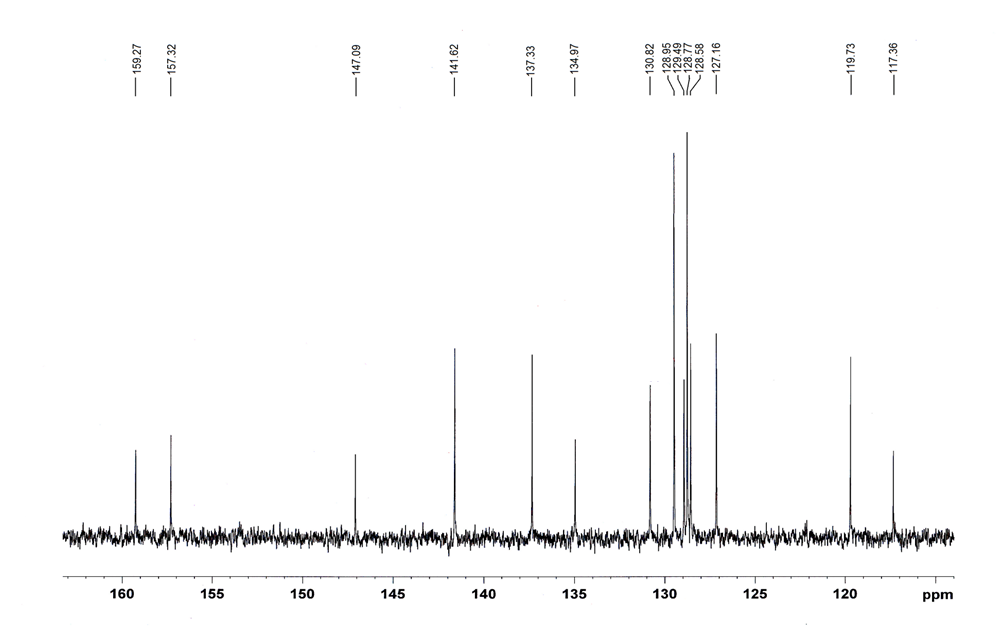


## ‎7.6 Enlarged ^13^C-NMR spectrum of **2g**

# The IR, 1H-NMR, 13C-NMR and HRMS spectra of product No. 2h


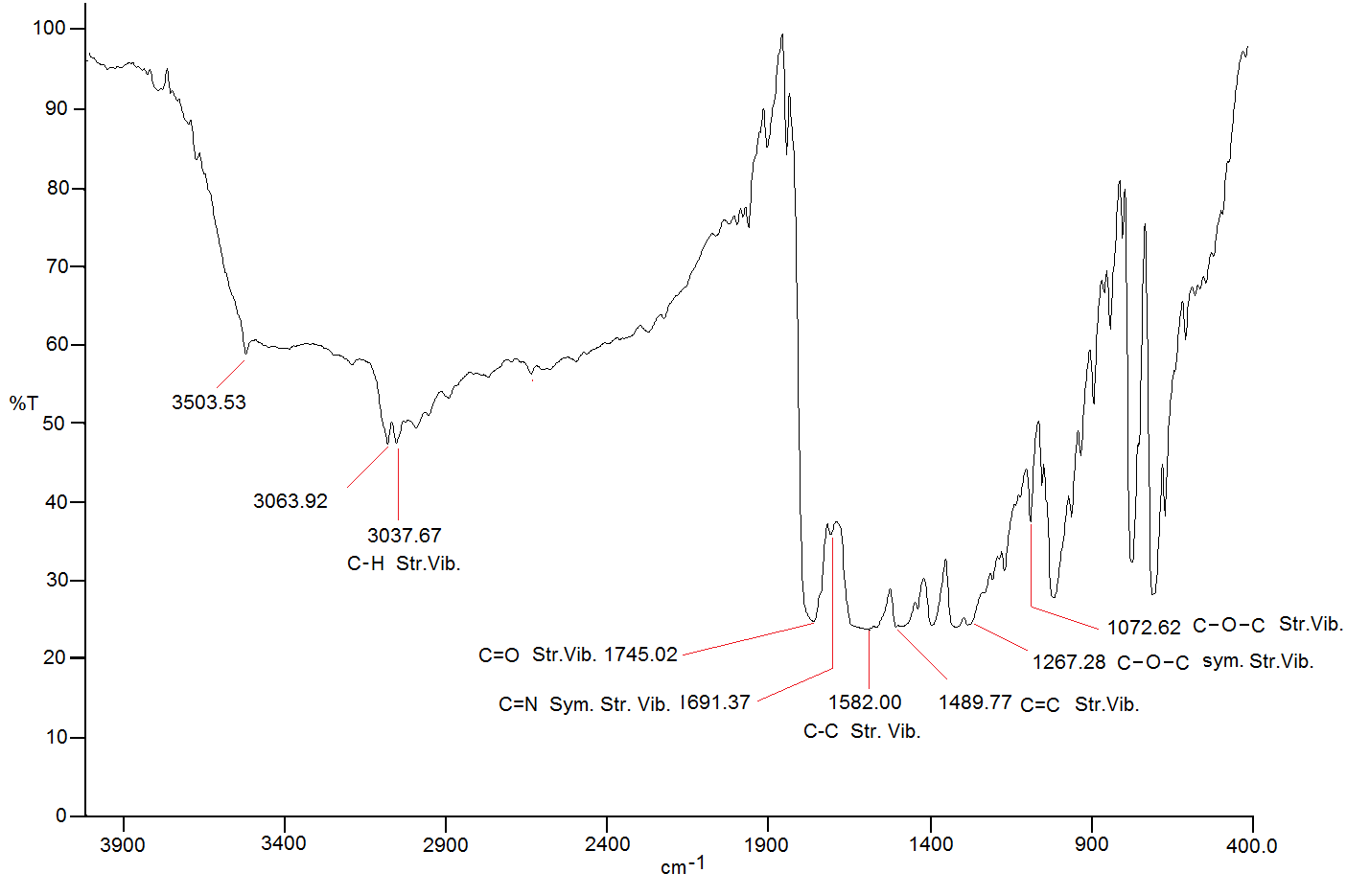


## ‎8.1 FT-IR spectrum of **2h**


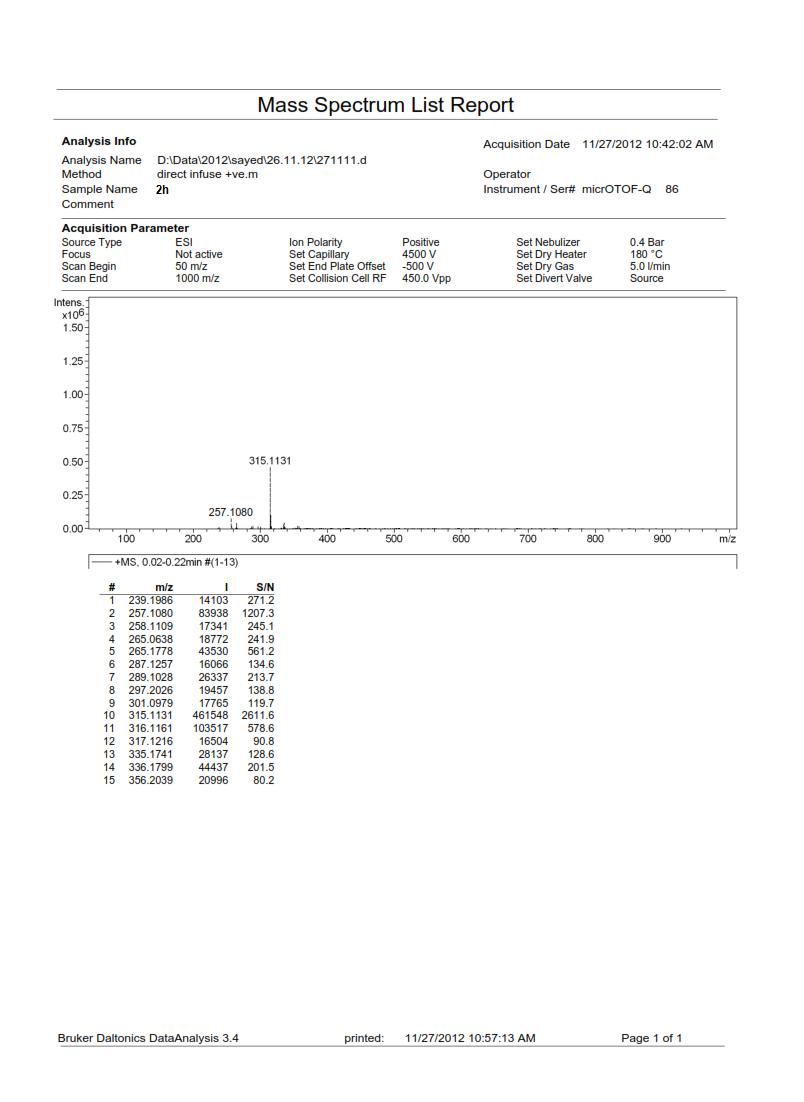


## ‎8.2 HRMS spectrum of **2h**


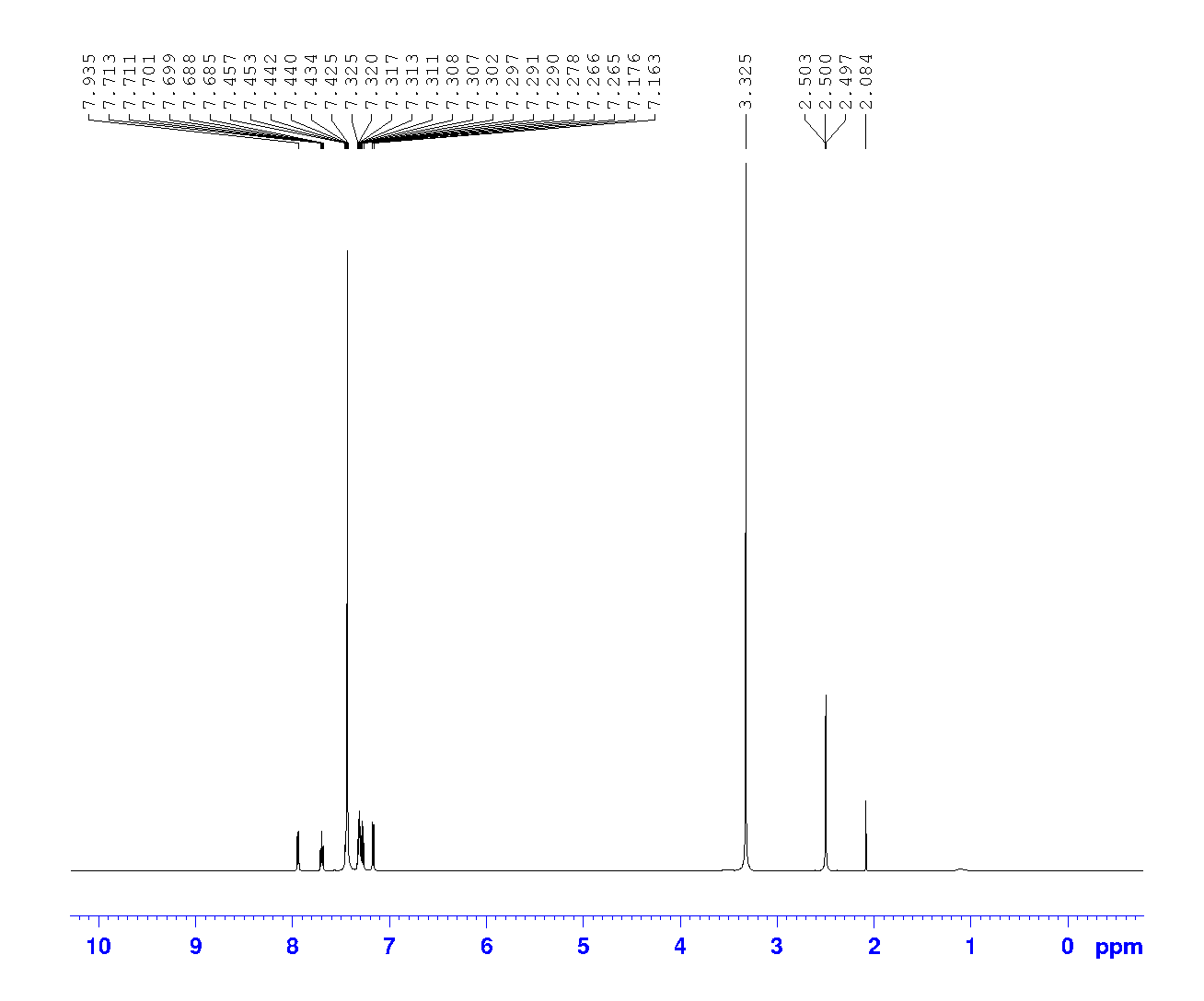


## ‎8.3 ^1^H-NMR spectrum of **2h**


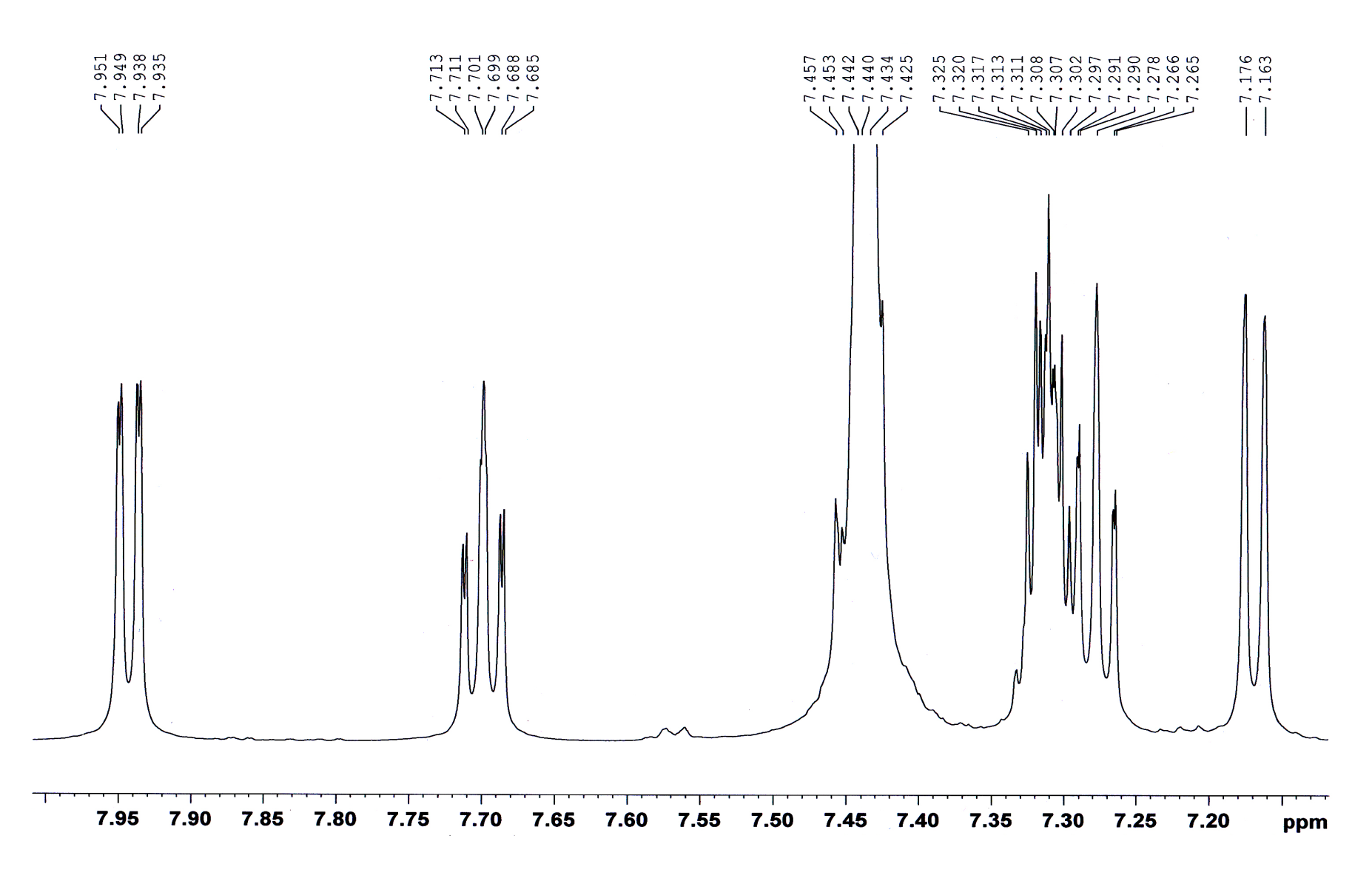


## ‎8.4 Enlarged ^1^H-NMR spectrum of **2h**


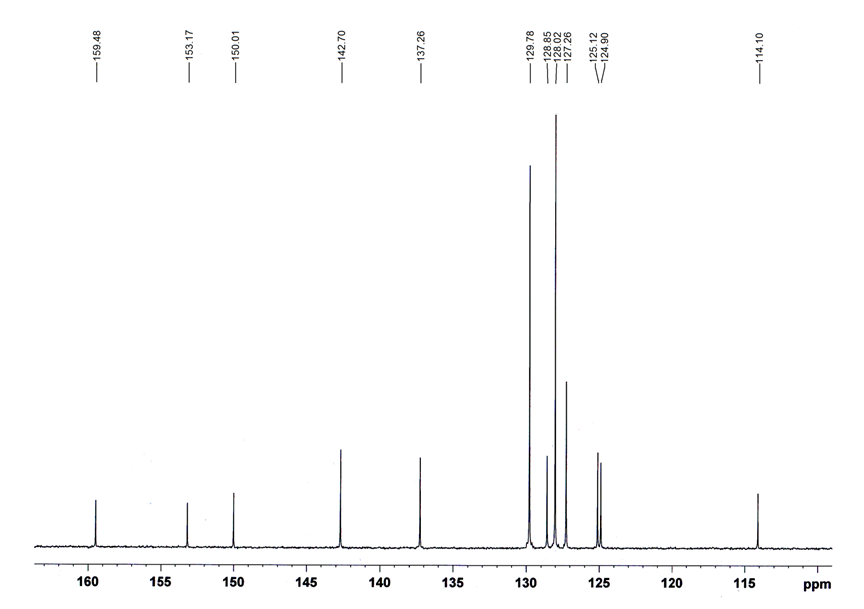


## ‎8.5 ^13^C-NMR spectrum of **2h**

# The IR, 1H-NMR, 13C-NMR and HRMS spectra of product No. 2i


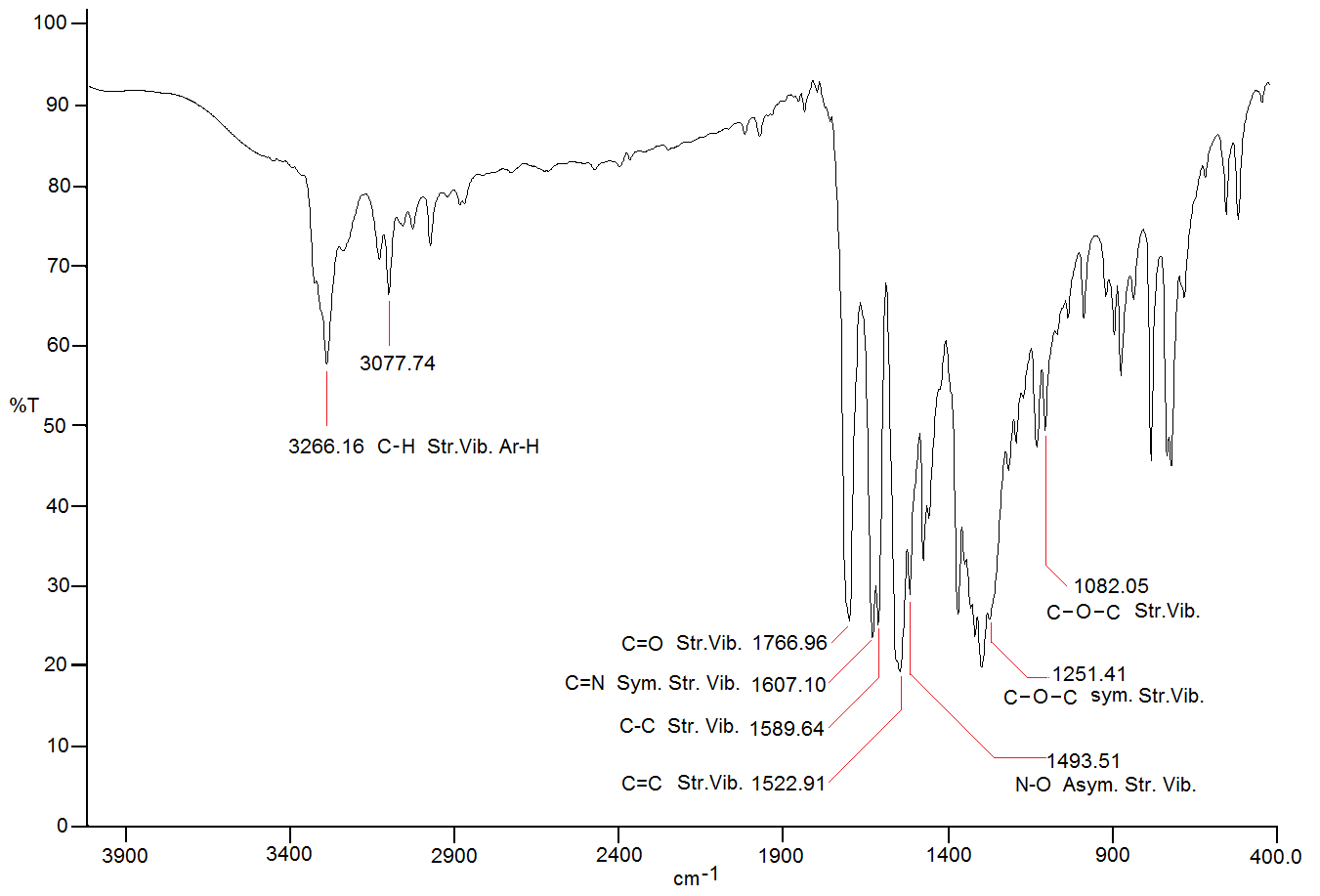


## ‎9.1 FT-IR spectrum of **2i**


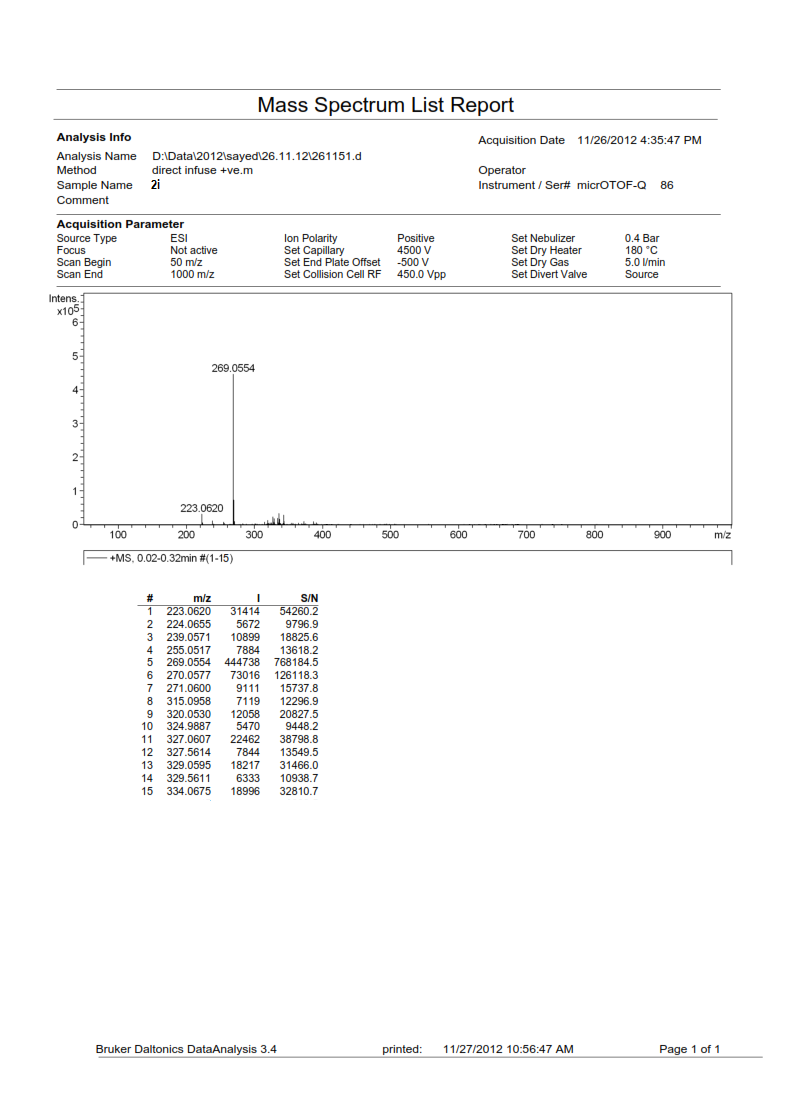


## ‎9.2 HRMS spectrum of **2i**


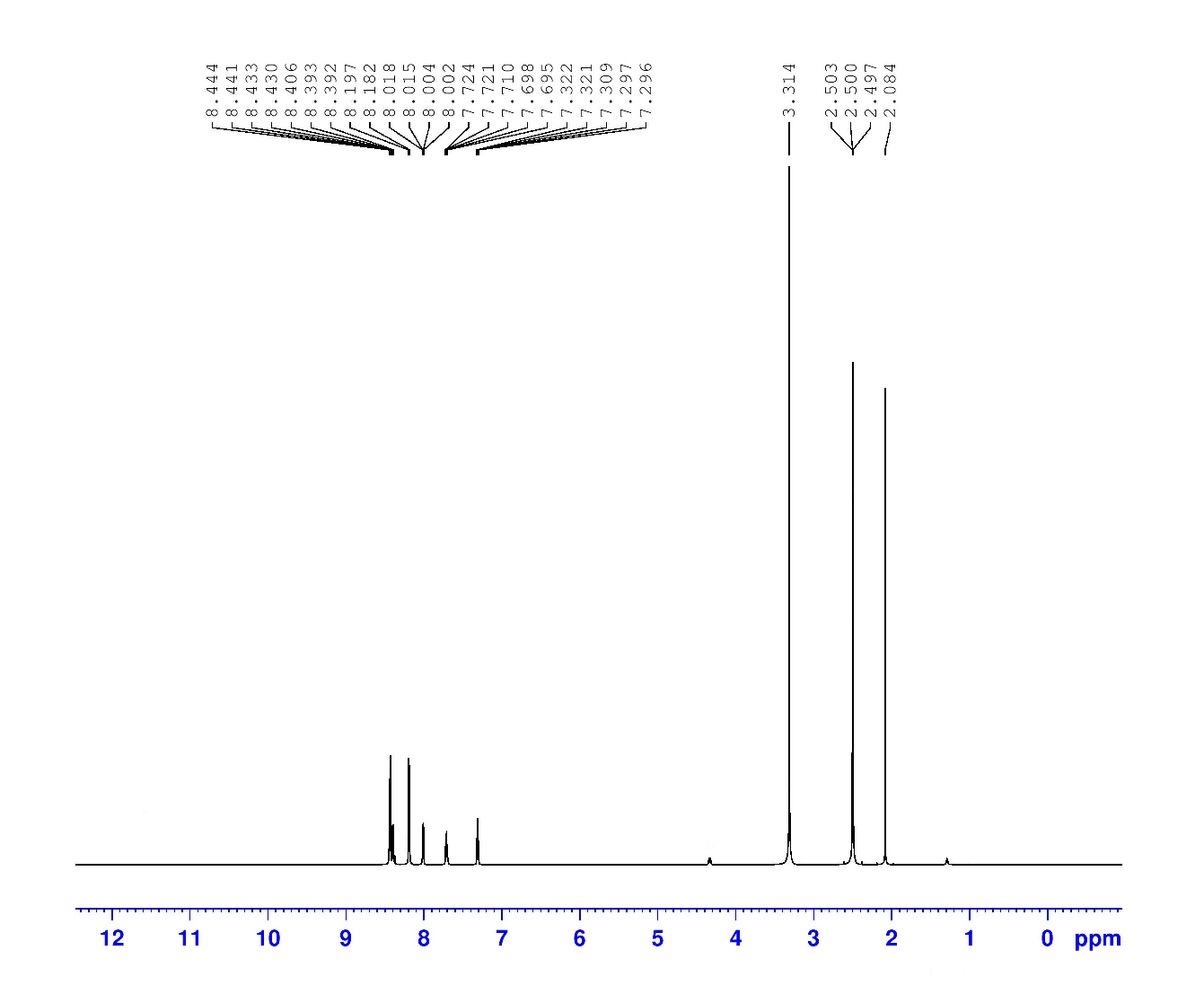


## ‎9.3 ^1^H-NMR spectrum of **2i**


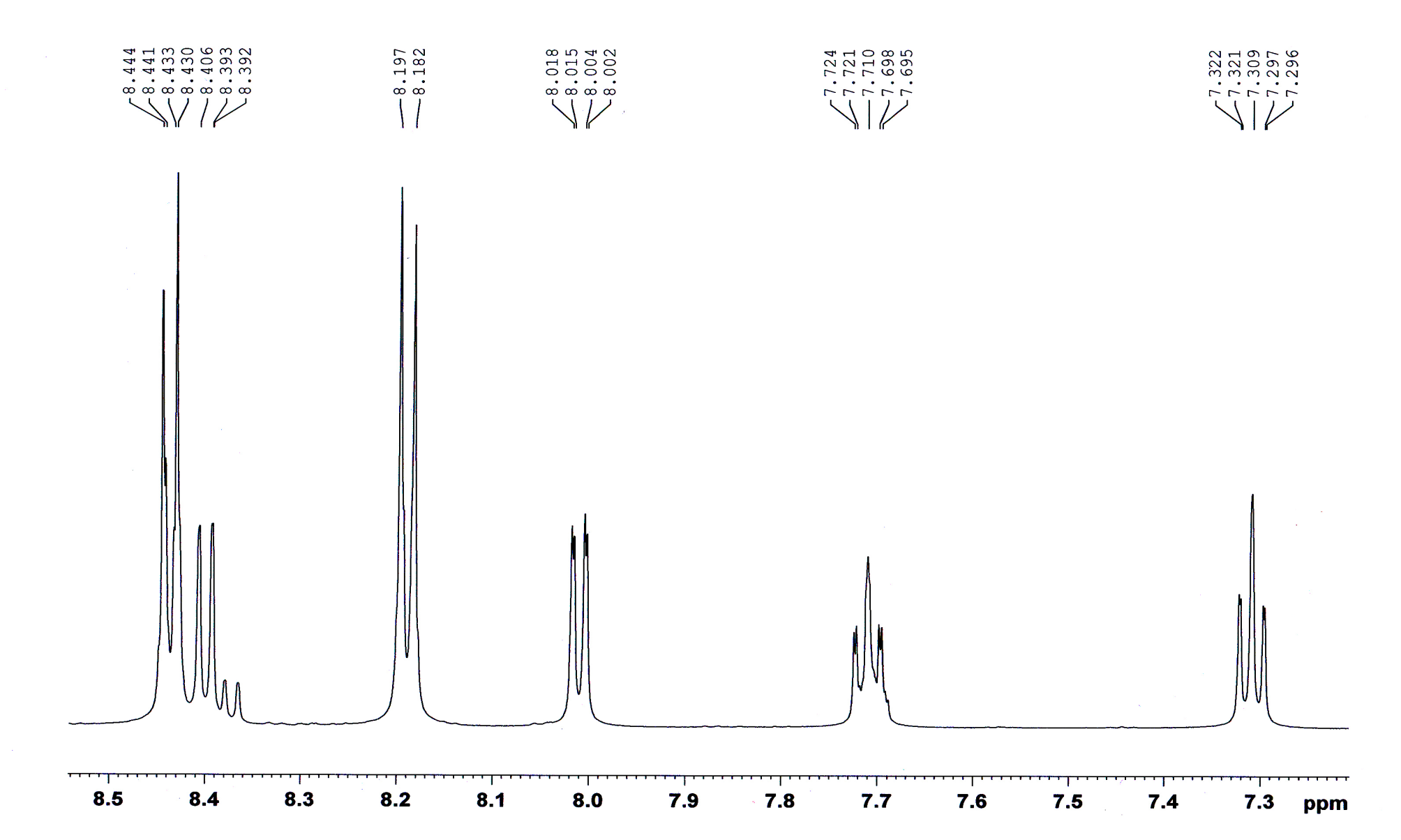


## ‎9.4 Enlarged ^1^H-NMR spectrum of **2i**


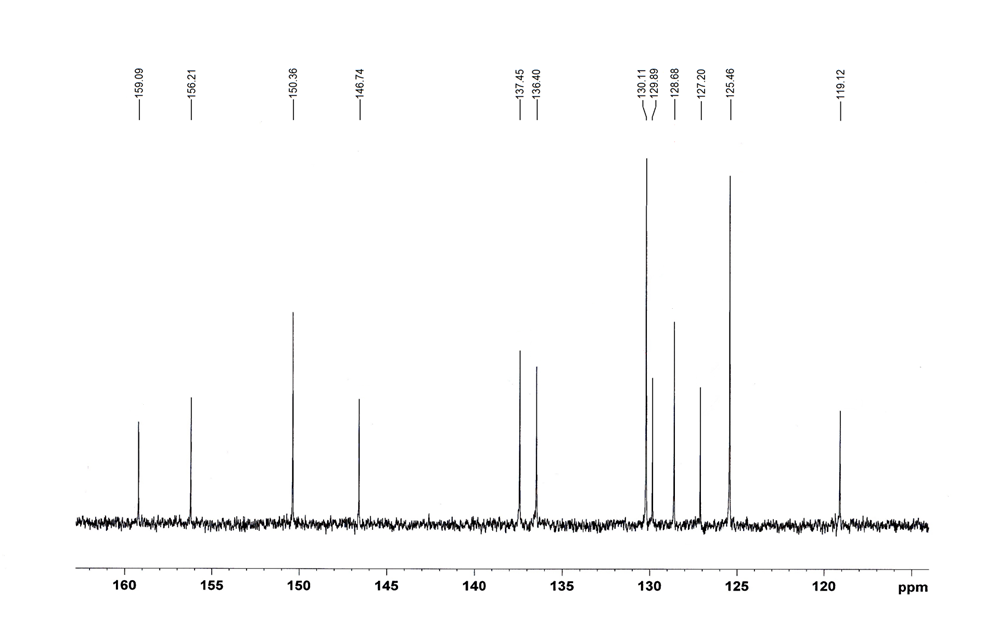


## ‎9.5 ^13^C-NMR spectrum of **2i**
